# Supplementary material for: The Mouse Gastrointestinal Bacteria Catalogue enables translation between the mouse and human gut microbiotas via functional mapping
Source: Cell Host Microbe. 2022 Jan 12;30(1):124–138.e8. doi: 10.1016/j.chom.2021.12.003 (PMC8763404; doi:10.1016/j.chom.2021.12.003)
Supplement: Document S1. Figures S1–S4 and Table S5 [file mmc1.pdf]

Cell Host & Microbe, Volume *30*

Supplemental information

The Mouse Gastrointestinal Bacteria Catalogue enables translation between the mouse and human gut microbiotas via functional mapping

Benjamin S. Beresford-Jones, Samuel C. Forster, Mark D. Stares, George Notley, Elisa Viciani, Hilary P. Browne, Daniel J. Boehmler, Amelia T. Soderholm, Nitin Kumar, Kevin Vervier, Justin R. Cross, Alexandre Almeida, Trevor D. Lawley, and Virginia A. Pedicord

Supplemental information:
The Mouse Gastrointestinal Bacteria Catalogue enables translation between the mouse and human gut microbiotas via functional mapping
Beresford-Jones et al.

Contents:
Supplementary Figures 1-4
Supplementary Table 5

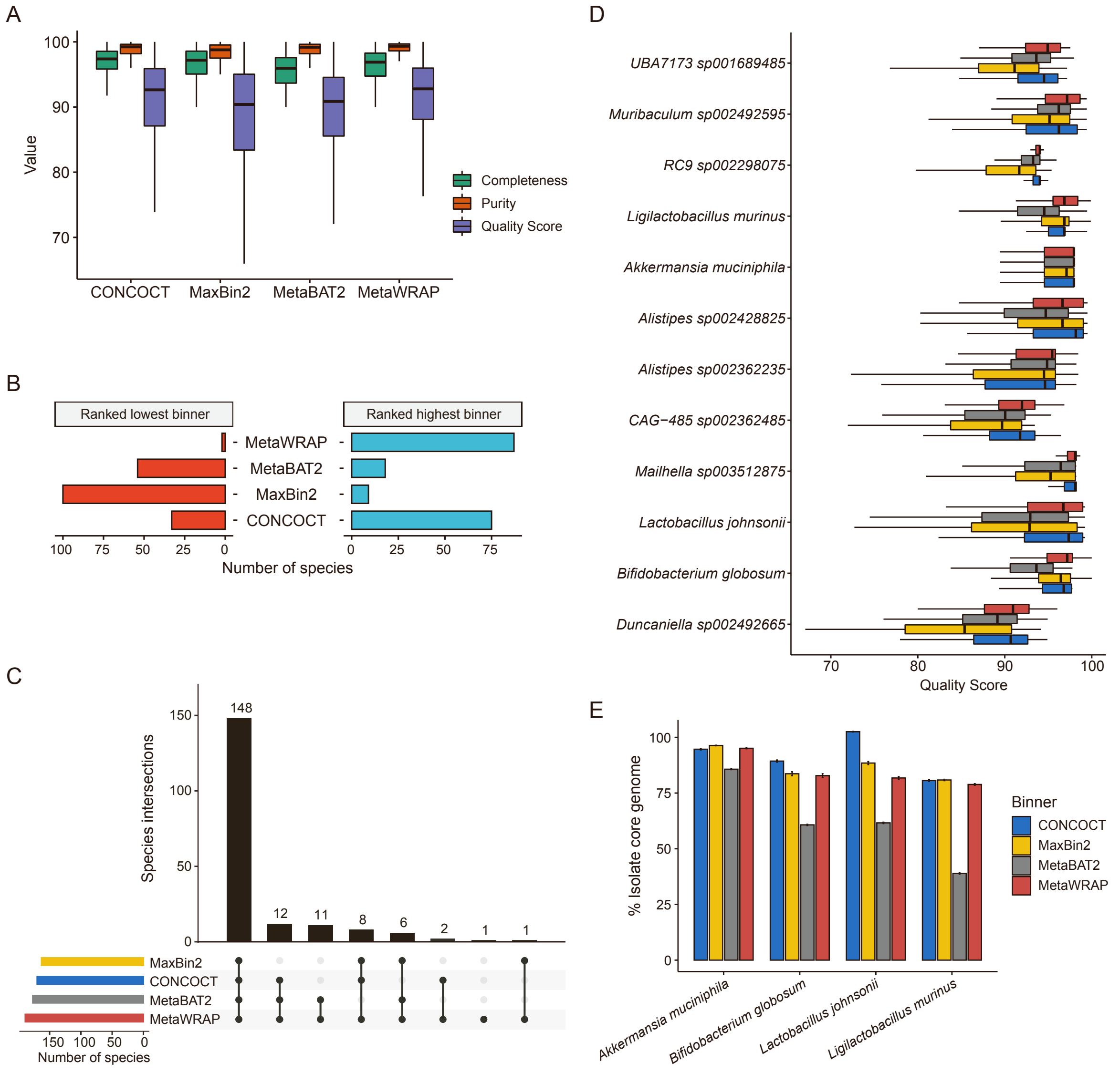

Supplementary Figure 1: Comparison of common binners for MAG synthesis, related to STAR methods. a) Quality scores of high-quality and medium-quality bins generated using the single binners MetaBAT2, MaxBin2, CONCOCT and hybrid bins from MetaWRAP (combined bins from all three single binners). b) Bars represent the number of species for which each binner generated the lowest quality bins ("Ranked lowest binner", red), and the highest quality ("Ranked highest binner", blue). c) UpSet plot illustrating the number of species represented by high-quality bins generated by each binner, and the species intersections between binners. d) MAG quality scores for the 12 most commonly binned species. e) Core genome size of commonly binned species when utilising different binners. Data represent the core genome size of MAG+isolate core genomes, compared to isolates alone. *A. muciniphila*, 100 iterations of 90 MAGs per binner and 136 isolate genomes; *B. globosum*, 100 iterations of 35 MAGs per binner and 62 isolate genomes; *L. johnsonii*, 100 iterations of 60 MAGs per binner and 54 isolate genomes; *L. murinus*, 100 iterations of 150 MAGs per binner and 58 isolate genomes. Aside from the medium quality MAG data in (a), only high-quality MAGs were used in these analyses.

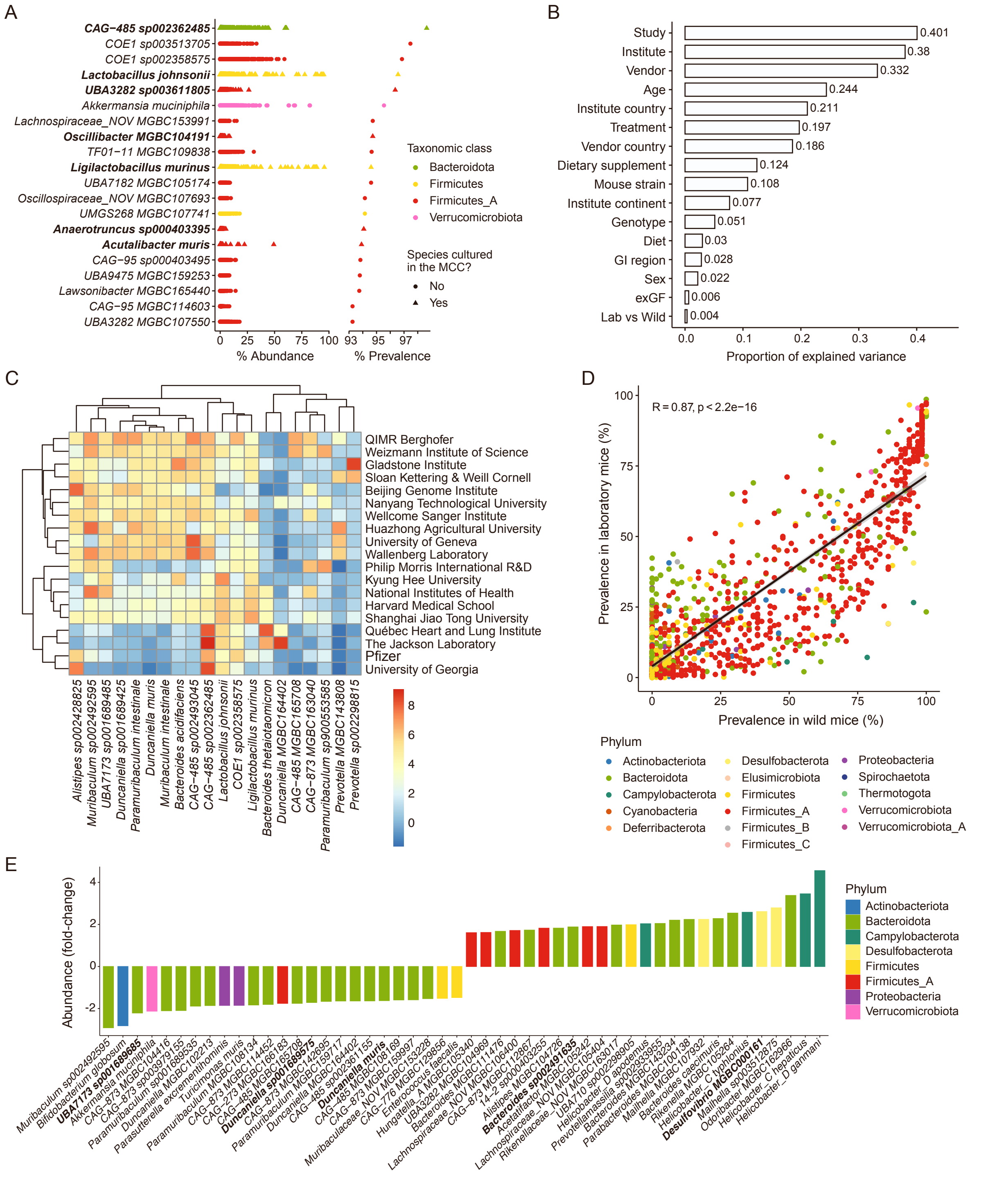

Supplementary Figure 2: The mouse gut microbiota between institutes and wild mice, related to STAR methods. a) Abundance and prevalence profiles of the 20 most prevalent species of the mouse gut microbiota across 2,446 samples. A species was determined as present in a sample if it was assigned ≥0.01% of classified reads. Point colour represents taxonomic phylum and point shapes and boldface labels indicate whether a species has been cultured as part of the MCC (triangles, bold) or not (circles). b) Proportion of variance explained (R2) by variables in the metadata using a permutational analysis of variance (PERMANOVA). All analyses were run with 999 permutations. Statistics for the PERMANOVA are provided in Table S7. c) Heatmap showing abundance of the top 20 most abundant species of the mouse microbiota across different institutes. Analyses include faecal samples from wildtype C57BL/6 "control" mice fed chow diets (n=432). Data are centre log-ratio

normalised read fractions, following Bayesian-multiplicative replacement of count zeros. d) Scatter plot comparing prevalence of species between untreated laboratory (n=1,065) and wild (n=65) mouse gut microbiotas. Each datapoint represents a mouse, and colour represents taxonomic phylum. Black line and shadow indicate linear regression line with 95% confidence interval ($r=0.87$, $p<2.2x10-16$). e) Fold-change in mean abundance of species in wild mice compared to untreated laboratory mice. Positive coefficient indicates enrichment in wild mice. Bar colour represents taxonomic phylum.

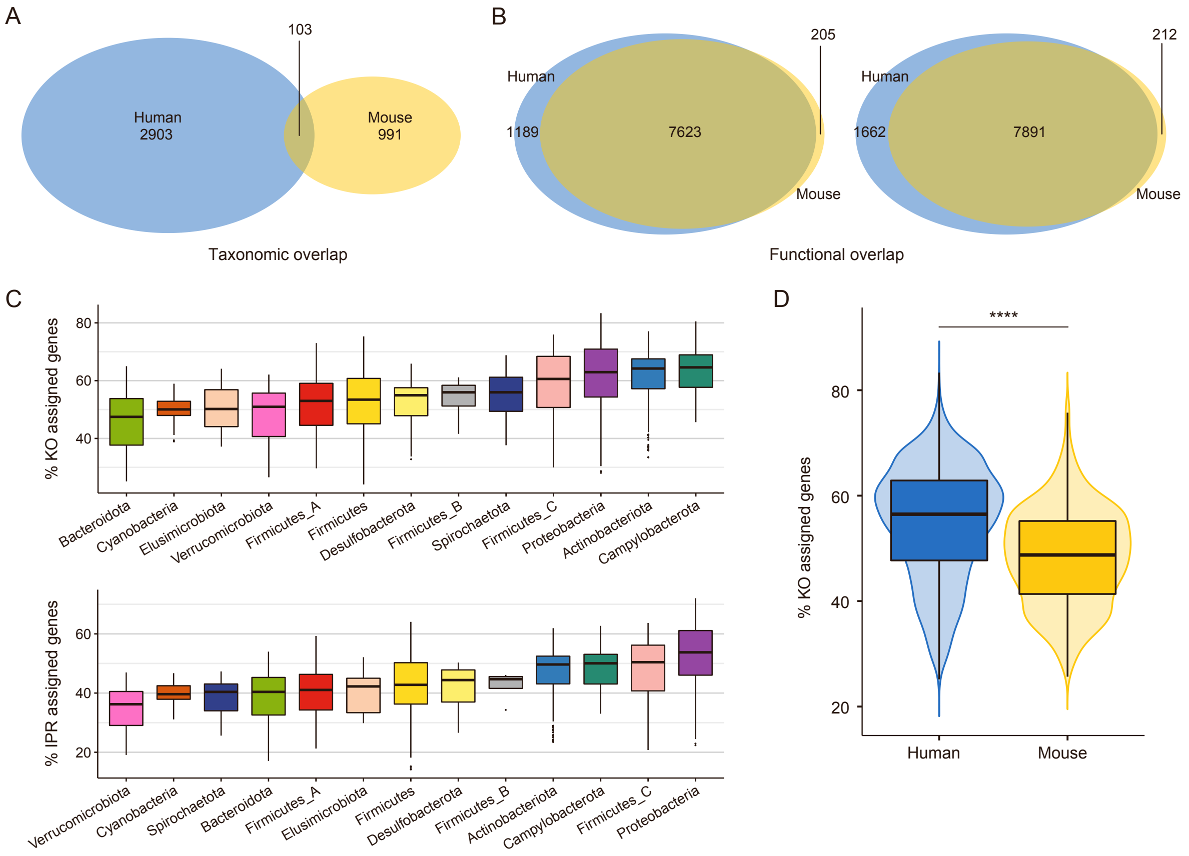

Supplementary Figure 3: Taxonomic and functional analyses of the human and mouse gut microbiotas, related to Figure 4. a) Venn diagram illustrating species sharing between human and mouse microbiotas. b) Venn diagrams illustrating the functional overlap of InterPro protein families (IPR; left) or KEGG Orthology (KO) groups (right) between all human (blue) and mouse (yellow) gut bacterial species. c) Functional annotation efficiency of bacterial pangenomes by taxonomic phylum for KO groups (top) and IPR families (bottom). Data represent the percentage of predicted protein-coding genes of each pangenome that could be assigned to an IPR or KO, coloured by phylum. d) Functional annotation efficiency of KO groups by host organism. A Wilcoxon signed-rank test was used to calculate statistical significance, ****P < 0.0001.

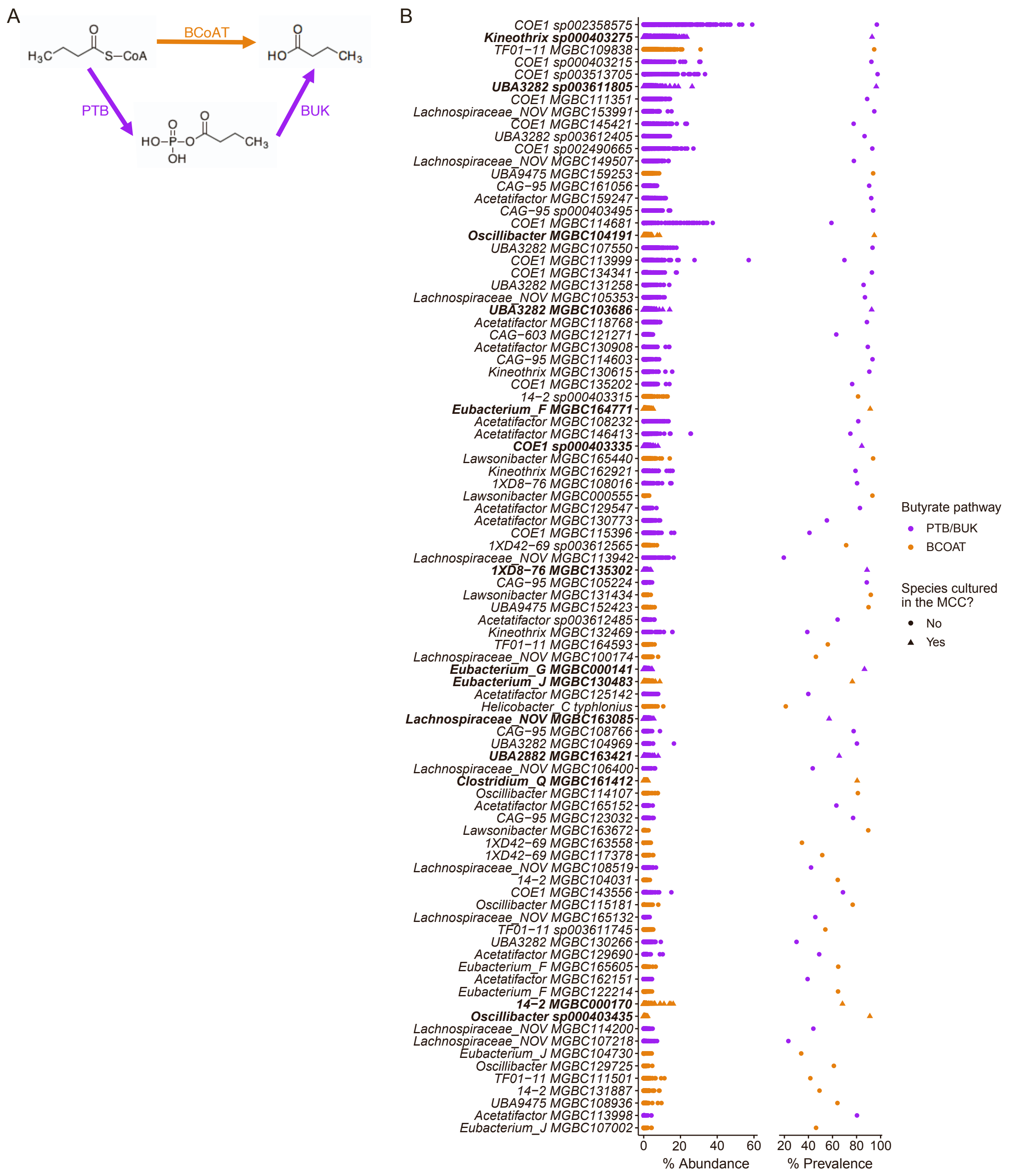

Supplementary Figure 4: Butyrate metabolism by species of the mouse gut microbiota, related to Figure 6. a) Schematic of the terminal pathways of butyrate synthesis by the gut microbiota. Butyrate CoA-transferase (BCoAT; orange); butyrate phosphotransferase/butyrate kinase (PTB/BUK; purple). b) Abundance and prevalence profiles of the 90 most abundant predicted butyrate producing species of the mouse gut microbiota. Point colour represents predicted encoded terminal butyrate pathway. Point shapes and boldface labels indicate whether a species has been cultured as part of the MCC (triangles, bold) or not (circles).

Supplementary Table 5: PERMANOVA statistics, related to STAR methods.
PERMANOVA comparing the extent to which factors in sample metadata explain variance between metagenomic samples.

Metadata factor	DegreesFreedom	SumsOfSquares	MeanSquares	F.Model	R2	P_value	Explanation of factor/variable:
Study	74	2055298	27774.3	21.42	0.40067	0.001	Study accession.
Institute	62	1949409	31442.1	23.56	0.38002	0.001	Experimental institute i.e., location of mice at time of sampling.
Vendor	57	1688944	29630.6	20.646	0.33217	0.001	Vendor's name with region if relevant e.g., Jackson, US vs Jackson, Denmark.
Age	54	1084273	20079.1	12.347	0.24407	0.001	Age at time of sampling.
Institute country	16	1074085	67130	40.29	0.21124	0.001	Country of the institute.
Treatment	58	1010917	17429.6	10.102	0.19728	0.001	What treatments did the mice receive e.g., control, high fat diet, specific drug treatment.
Vendor country	14	932012	66572	38.714	0.18555	0.001	Country of the vendor.
Dietary Supplement	30	635939	21198	11.392	0.1241	0.001	Dietary supplement, including in the drinking water e.g., glucose, NSAIDs, or DSS.
Mouse strain	20	553619	27681	14.671	0.10806	0.001	Strain of mice e.g., C57BL/6J vs C57BL/6NTac
Institute continent	3	394842	131614	67.88	0.07697	0.001	Continent of the institute.
Genotype	17	263389	15493.5	7.7309	0.05141	0.001	Genotype: wildtype, or specific knockout e.g., IL-10 deficient.
Diet	10	152023	15202.3	7.437	0.03	0.001	Diet: chow, high fat, low fibre, etc.
Sample source/GI location	10	143611	14361.1	7.0171	0.02825	0.001	Sampling location e.g., faeces, caecum, small intestine.
Sex	1	94158	94158	45.948	0.02201	0.001	Sex of mice.
exGF	1	32620	32620	15.641	0.00636	0.001	Endogenous (SPF) or reconstituted (ExGF) microbiota.
Lab vs Wild	1	18098	18098.1	8.6532	0.00353	0.001	Laboratory vs wild mice, reflecting their faecal origins e.g., in the case of ExGF mice

Technical features:	Permutation:	Free
	Number of permutations:	999
