# Supplementary material for: The Mouse Gastrointestinal Bacteria Catalogue enables translation between the mouse and human gut microbiotas via functional mapping
Source: Cell Host Microbe. 2022 Jan 12;30(1):124–138.e8. doi: 10.1016/j.chom.2021.12.003 (PMC8763404; doi:10.1016/j.chom.2021.12.003)
Supplement: Document S2. Article plus supplemental information [file mmc12.pdf]

Resource

Cell Host & Microbe

The Mouse Gastrointestinal Bacteria Catalogue enables translation between the mouse and human gut microbiotas via functional mapping

Graphical abstract

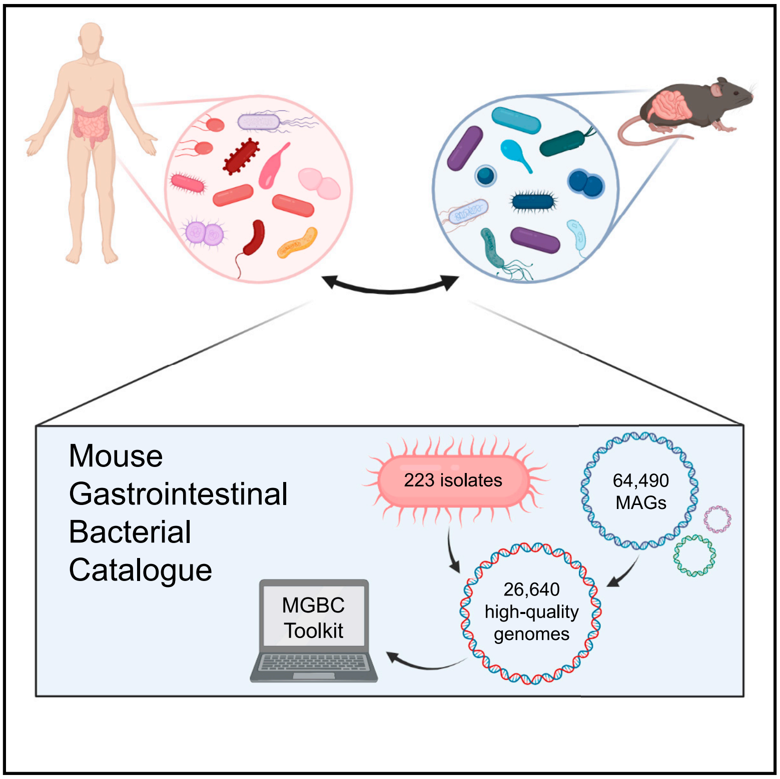

Highlights

- MGBC allows functional translation between human and mouse microbiotas
- Previously uncultured isolates expand culture collection of mouse gut microbes
- Bioinformatic toolkit maps taxonomic locations of microbial functions of interest

Authors

Benjamin S. Beresford-Jones, Samuel C. Forster, Mark D. Stares, ..., Alexandre Almeida, Trevor D. Lawley, Virginia A. Pedicord

Correspondence

tl2@sanger.ac.uk (T.D.L.),
vap33@cam.ac.uk (V.A.P.)

In brief

Beresford-Jones et al. find that while less than 3% of bacterial species are shared between human and mouse gut microbiotas, they can identify the closest functionally related species between these host-specific microbiotas using the bacterial genome catalog they developed. This will facilitate translation of microbiota-related research between humans and mice.

Beresford-Jones et al., 2022, Cell Host & Microbe *30*, 124–138
January 12, 2022 © 2021 The Authors. Published by Elsevier Inc.
https://doi.org/10.1016/j.chom.2021.12.003

CellPress
OPEN ACCESS

Cell Host & Microbe

Resource

The Mouse Gastrointestinal Bacteria Catalogue enables translation between the mouse and human gut microbiotas via functional mapping

Benjamin S. Beresford-Jones,[1,2] Samuel C. Forster,[3] Mark D. Stares,[3] George Notley,[3] Elisa Viciani,[3] Hilary P. Browne,[3] Daniel J. Boehmler,[4] Amelia T. Soderholm,[1,2] Nitin Kumar,[3] Kevin Vervier,[3] Justin R. Cross,[4] Alexandre Almeida,[3,5] Trevor D. Lawley,[3,6,*] and Virginia A. Pedicord[1,2,6,7,*]
[1]Cambridge Institute of Therapeutic Immunology and Infectious Disease, Jeffrey Cheah Biomedical Centre, Cambridge Biomedical Campus, Cambridge, UK
[2]Department of Medicine, University of Cambridge School of Clinical Medicine, Cambridge Biomedical Campus, Cambridge, UK
[3]Wellcome Sanger Institute, Wellcome Genome Campus, Hinxton, UK
[4]Donald B. and Catherine C. Marron Cancer Metabolism Center, Memorial Sloan Kettering Cancer Center, New York, NY, USA
[5]European Bioinformatics Institute, EMBL-EBI, Wellcome Genome Campus, Hinxton, UK
[6]These authors contributed equally
[7]Lead contact
*Correspondence: tl2@sanger.ac.uk (T.D.L.), vap33@cam.ac.uk (V.A.P.)
https://doi.org/10.1016/j.chom.2021.12.003

SUMMARY

Human health and disease have increasingly been shown to be impacted by the gut microbiota, and mouse models are essential for investigating these effects. However, the compositions of human and mouse gut microbiotas are distinct, limiting translation of microbiota research between these hosts. To address this, we constructed the Mouse Gastrointestinal Bacteria Catalogue (MGBC), a repository of 26,640 high-quality mouse microbiota-derived bacterial genomes. This catalog enables species-level analyses for mapping functions of interest and identifying functionally equivalent taxa between the microbiotas of humans and mice. We have complemented this with a publicly deposited collection of 223 bacterial isolates, including 62 previously uncultured species, to facilitate experimental investigation of individual commensal bacteria functions *in vitro* and *in vivo*. Together, these resources provide the ability to identify and test functionally equivalent members of the host-specific gut microbiotas of humans and mice and support the informed use of mouse models in human microbiota research.

INTRODUCTION

The mammalian gastrointestinal tract hosts trillions of bacteria, known as the gut microbiota, that actively impact the health of the host. Variations in this bacterial ecosystem are associated with susceptibility to and outcomes of many human diseases (Armour et al., 2019), from adverse nutritional states (Chen et al., 2021; Sonnenburg and Bäckhed, 2016) and autoimmunity (Li et al., 2018) to neurological pathologies (Cryan et al., 2020) and infection (Libertucci and Young, 2019). In order to characterize these microbial associations for therapeutic benefit, it is necessary to establish causal relationships between microbial factors and phenotypes (Neville et al., 2018). To this end, mouse models are essential tools in microbiota research, allowing controlled experimental studies in a physiologically and genetically tractable system.

A recognized limitation in using mice to study the human gut microbiota is that few bacterial species are shared between the gastrointestinal tracts of humans and mice (Chung et al., 2012; Xiao et al., 2015). In addition, many mouse-derived species remain unidentified or uncharacterized, hindering translation of microbiota research between hosts. One approach that has been used to improve the utility of mice for human microbiota research is the colonization of mice with human microbiotas (Park and Im, 2020). However, while it has been reported that the microbiotas of humans and mice are functionally comparable (Liu et al., 2020; Xiao et al., 2015), mice treated with a human microbiota exhibit compromised immune maturation and performance compared with mice harboring a mouse-derived microbiota (Chung et al., 2012; Lundberg et al., 2020), resulting in differences in susceptibility to infectious (Chung et al., 2012) and inflammatory (Surana and Kasper, 2017) diseases. This may be due in part to an inability of some human microbes to colonize well in the mouse gut (Aluthge et al., 2020; Lundberg et al., 2020). It is, therefore, likely that studying commensal bacteria in their endogenous hosts is the most physiologically valid approach. This requires the ability to identify functionally equivalent species between host microbiotas to perform mechanistic experimental studies in mice and translate microbiota findings between humans and mice.

 © 2021 The Authors. Published by Elsevier Inc.
This is an open access article under the CC BY license (http://creativecommons.org/licenses/by/4.0/).

CellPress
OPEN ACCESS

Accurate species-resolved functional analyses require comprehensive and complete genomes (Meziti et al., 2021; Shaiber and Eren, 2019), and both culture-dependent and culture-independent approaches have been implemented to characterize the human (Almeida et al., 2019; Forster et al., 2019; Lagier et al., 2016; Nayfach et al., 2019; Pasolli et al., 2019; Poyet et al., 2019; Zou et al., 2019) and mouse (Lagkouvardos et al., 2019, 2016; Lesker et al., 2020; Liu et al., 2020) gut microbiotas. Cultured isolates provide highly complete reference genomes and are essential for experimental validation of correlative findings (Neville et al., 2018; Surana and Kasper, 2017). Owing to current challenges in culturing the complete diversity of the gut microbiota, culture-independent approaches, most commonly metagenome-assembled genome (MAG) reconstruction from shotgun metagenome samples, have been leveraged to generate genomes for uncultured species and improve coverage in microbiome analyses (Forster et al., 2019). Gene catalogs have also been generated for improving metagenome analyses (Li et al., 2014; Qin et al., 2010; Xiao et al., 2015; Zhu et al., 2021); however, as these catalogs do not link genes to their genomes of origin, they cannot be used for genome-resolved functional analyses (Almeida et al., 2021).

Here, we complement high-throughput culturing of mouse gut bacteria with large-scale MAG synthesis to produce a comprehensive isolate and genome repository, the Mouse Gastrointestinal Bacteria Catalogue (MGBC). The MGBC includes 26,640 non-redundant, high-quality bacterial genomes representing 1,094 species and a publicly deposited collection of 223 cultured isolates for 132 species, including 62 species with no previously cultured representative. Through species-resolved functional mapping of the gut microbiotas of humans and mice, this resource enables the identification of the closest functionally related bacterial species between hosts and provides access to the taxonomic locations of shared functions, allowing the application and investigation of microbiota discoveries between hosts.

RESULTS

Expanding the cultured diversity of the mouse gut microbiota

Bacterial culturing and isolate biobanking are essential for research and biotechnology applications by enabling the validation of *in silico* findings and investigation of underlying biological mechanisms. Nearly 10,000 isolate genomes exist for human-derived commensal bacteria (Almeida et al., 2021; Poyet et al., 2019), but fewer than 400 have been published for the mouse. To begin to address this disparity, we performed high-throughput culturing of feces from 30 conventionally housed, specific-pathogen-free (SPF) laboratory mice from the Wellcome Sanger Institute. We cultured and whole-genome sequenced 288 bacterial strains, of which 276 genomes passed our stringent quality control criteria to be included in our mouse culture collection (MCC; Figure 1A; Table S1). These isolates represent 132 species across 67 genera, 25 families, and 6 phyla and include 62 taxa with no previously cultured murine representative (i.e., they share <95% average nucleotide identity (ANI) with the closest related genome). The majority of these additional cultured bacterial species belong to the phylum Firmicutes_A (54/62), with the remaining isolates from the Bacteroidota (4/62), Firmicutes (3/62), and Desulfobacterota (1/62) phyla (Figure 1B). These previously uncultured isolates increase the cultured microbial diversity of the mouse gut microbiota by 32.5% at the species level and provide the first cultured representatives for 10 genera, as well as the first mouse-derived representative of the phylum Desulfobacterota. In order to establish the prevalence of these species, we compiled 2,398 publicly available and sequenced 48 new mouse gut metagenomes, representing mice from 63 institutes across 17 countries (Table S2). Using this global compilation, we found that 20 of our additional species are present in more than 80% of samples, while 39 were present in over 50% (Figures 1B and 1C), indicating that our novel cultured isolates represent highly prevalent species of the mouse gut microbiota. To make these bacteria easily accessible to the scientific community, the 223 isolates that were successfully cryopreserved, are currently available upon request from the Wellcome Sanger Institute and are additionally being deposited with the German Collection of Microorganisms and Cell Cultures (DSMZ).

In addition, we compiled 319 publicly available mouse-derived bacterial isolate genomes from the NCBI, including genomes from the mouse intestinal Bacterial Collection (miBC, n = 53) (Lagkouvardos et al., 2016) and the mouse Gut Microbial Biobank (mGMB, n = 120) (Liu et al., 2020), among other studies (Table S1). Following genome quality filtering, 288 public genomes were combined with our MCC to yield 564 cultured isolate genomes, representing 253 species that we included in our MGBC genome repository.

Construction of the MGBC

Multiple studies have resulted in over 100,000 high-quality MAGs for species of the human gut microbiota (Almeida et al., 2021), but fewer than 8,000 high-quality MAGs have been generated for the mouse (Lesker et al., 2020). To facilitate translation between the human and mouse gut microbiotas, we sought to construct a comprehensive, genome-resolved catalog for mouse gut bacterial species that would allow species-level functional comparisons with the gut bacteria of human hosts. As highly complete genomes are required for accurate functional analyses and identification of functionally equivalent species, we first compared binning methods for MAG generation using metrics of genome quality and completeness (Parks et al., 2015). Compared with using MetaBAT2, MaxBin2, or CONCOCT alone, the MetaWRAP pipeline (Uritskiy et al., 2018) yielded bins with the highest quality scores (Figure S1A) and generated the highest quality MAGs for the most species (Figures S1B and S1C). Considering the most dominantly binned species of the mouse gut microbiota, MetaWRAP consistently yielded the highest quality MAGs (Figure S1D) and conserved a large proportion of the core genome (Mdn [Inter-Quartile Range (IQR)]: *Lm* = 79.04% [77.48–80.19]; *Lj* = 81.62% [79.84–81.77]; *Bg* = 83.35% [80.19–86.47]; *Am* = 95.23% [94.47–95.87]; Figure S1E). Based on these results, we employed MetaWRAP to generate MAGs for the MGBC.

To produce a globally representative genome collection, we assembled and binned publicly available mouse gut metagenomes from the European Nucleotide Archive (ENA) in addition to the 48 new fecal samples that were sequenced from mice at

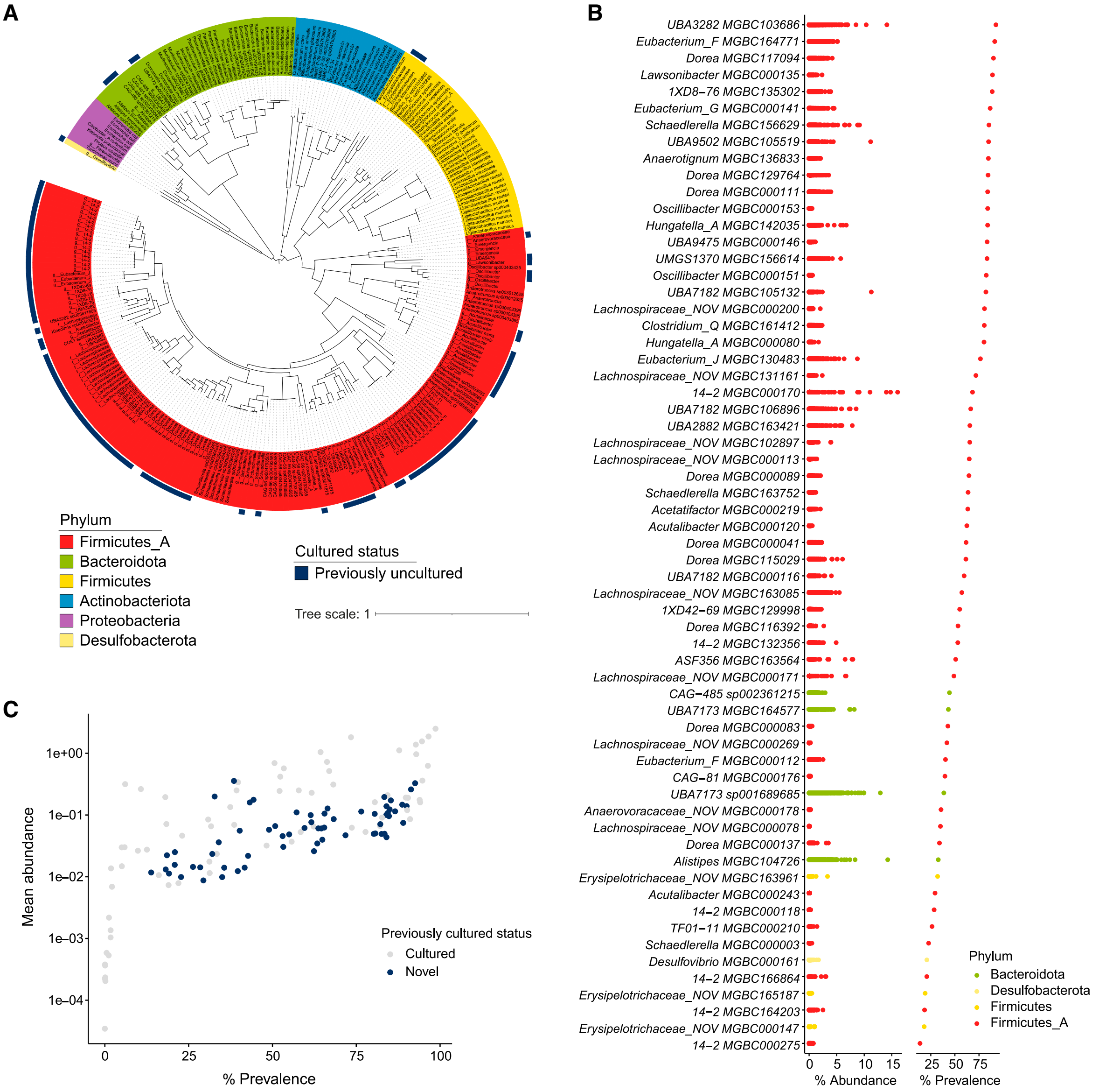

Figure 1. Isolates of the mouse culture collection

(A) Maximum likelihood phylogenetic tree of the 276 bacterial isolate genomes of the MCC. Genome labels indicate genome taxon as assigned by GTDB-Tk; where a genome could not be assigned at species level, lowest taxonomic rank is indicated. Labels are colored by phylum, and the outer ring indicates genomes with no previously cultured representative. Tree distances were calculated from an alignment of 120 core genes using the BLOSUM45 amino acid similarity matrix.

(B) Abundance and prevalence profiles for the 62 previously uncultured species of the MCC based on 2,446 mouse gut metagenomes. Each datapoint represents the percentage of reads assigned to a species for a single sample. Prevalence is calculated as the percentage of samples with species abundance $\geq$0.01%. Colors represent phyla.

(C) Scatterplot of mean abundance against prevalence for all 132 species of the MCC. Color represents prior cultured status.

the Wellcome Sanger Institute and the Babraham Institute. Of these metagenomes, 2,286 samples yielded $\geq$1 high-quality MAGs per sample; these samples represented 71 studies and 13 strains of mice from 58 institutes across 17 countries and 4 continents. In total, these efforts yielded 64,490 medium-plus quality MAGs (Table S3), of which 35,361 were defined as high-quality in line with previous studies (Almeida et al., 2021; Pasolli et al., 2019). To remove virtually identical genomes from our collection, we dereplicated our isolate genomes and MAGs using a Mash distance (Ondov et al., 2016) of 0.001 (equivalent to 99.9% ANI) to yield 26,640 high-quality, non-redundant bacterial genomes for the MGBC (Figure 2A; Table S3). Of all the species cultured in the MCC (n = 132), 74.2% are represented by at least one high-quality MAG. Isolate genomes were significantly more complete than MAGs (98.9% [98.3–99.4] versus 96.9% [94.8–98.3], Mdn [IQR]; p < 0.0001) but were equivalent in terms of contamination (0.77% [0.19–1.72] versus 0.64% [0.23–1.35]; p = 0.22). Overall, the quality scores of isolate genomes were

CellPress
OPEN ACCESS

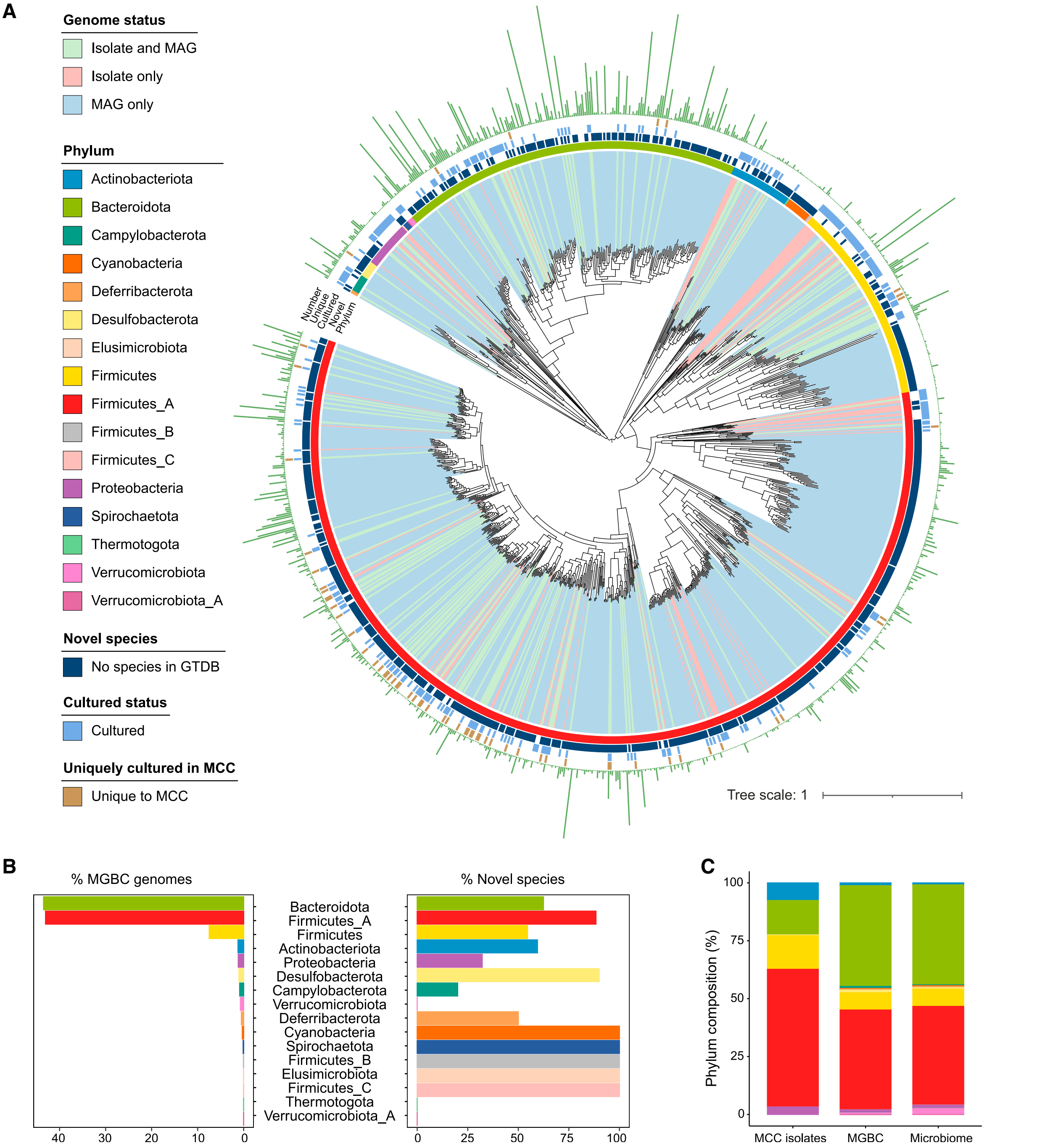

Figure 2. Genomes of the Mouse Gastrointestinal Bacteria Catalogue

(A) Maximum likelihood tree of representative genomes for the 1,094 species of the MGBC. Color range indicates whether a species cluster is represented by MAGs only (light red), isolates only (light green), or both (light blue). For each species, the innermost color ring represents phylum, the second ring indicates species that could not be assigned at a species level by GTDB-Tk (dark blue), the third ring denotes cultured status of each species (blue), and the outer ring indicates the 62 species that have been uniquely cultured in the MCC (brown). The circumferential bar plot (green) illustrates the number of high-quality genomes representing each species in the MGBC. Tree distances were calculated from an alignment of 120 core genes using the BLOSUM45 amino acid similarity matrix.

(B) Phylum-level distribution of the 26,640 high-quality genomes of the MGBC (left) and percentage of species clusters not assigned to a species-level taxonomy by GTDB-Tk (right).

(C) Stacked bar plots comparing the phylum-level composition of the 276 MCC isolates (MCC isolates) and the 26,640 genomes of the MGBC with the average mouse microbiome (microbiome; n = 2,446). The distributions of each stacked bar were compared using a chi-square test for Independence. MCC, microbiome ($p = 0.015$, significantly different); MGBC, microbiome ($p = 1$, not significantly different).

higher than those of MAGs (95.2 [89.6–98.0] versus 93.0 [88.4–96.1]; $p < 0.0001$). Notably, despite the number of samples considered, no bins were generated for the domain Archaea, potentially due to very low abundance of this domain in the mouse gastrointestinal tract (Zhu et al., 2021).

The MGBC represents 1,094 species of the mouse gastrointestinal tract across 16 phyla (Figure 2A). Only 23.1% of these species have cultured representatives, of which 24.5% are contributed uniquely by our MCC. Only 23.5% of species could be assigned to a species-level taxonomy by the Genome Taxonomy Database Toolkit (GTDB-Tk) (Chaumeil et al., 2019), with 61.3% unassigned at the species level, 14.0% at the genus level, and 1.2% at the family level. Species of the Cyanobacteria, Spirochaetota, Firmicutes_B, Elusimicrobiota, and Firmicutes_C were represented entirely by taxa unassigned at the species level (Figure 2B). In contrast to the MCC, the MGBC is highly representative of the average mouse microbiome at the phylum-level (Figure 2C). These findings underpin our use of both isolates and MAGs to produce a comprehensive high-quality genome catalog of the mouse gut microbiota to provide a framework for species-resolved functional comparisons.

Benchmarking the MGBC against current mouse microbiota resources

To maximize the reliability and accuracy of our analyses, we curated only our high-quality genomes into the MGBC (Figures 3A and 3B). To benchmark our resource against currently available mouse microbiota resources, we compared the genomes of the MGBC with the high-quality genomes of the integrated mouse gut metagenome catalog (iMGMC) (Lesker et al., 2020). The 8,509 high-quality genomes of the iMGMC represent 805 species, of which 670 (83.2%) are shared with the MGBC (Figure 3C). Comparing the representative genomes for these shared species, 71.8% of species representatives from the MGBC had a higher quality score than their equivalent in the iMGMC (Figure 3D), while 14.6% were equal in quality. The MGBC achieves significantly higher levels of metagenome read classification of independent samples, classifying 90.5% of reads on average, 8.4% more than with the iMGMC, and 56.9% more than with the medium-plus quality co-abundance gene groups (CAGs) of the MGCv1 (Xiao et al., 2015) (Figure 3E). The MGBC represents an additional 407 species compared with the iMGMC, expanding the known taxonomic diversity of the mouse gut microbiota by 77 genera (30.6% increase), 25 families (31.8% increase), 15 orders (34.1% increase), 3 classes (15% increase), and 2 phyla (12.5% increase). Therefore, this resource, provides improved coverage and quality of representation of the mouse gut microbiota compared with the previous mouse microbiota resources, better facilitating species-resolved functional analyses and improving overall characterization of the mouse gut microbiota.

Previous studies have performed taxonomic profiling of the mouse gut microbiota using alternative methods and/or smaller sample sizes (Lesker et al., 2020; Xiao et al., 2015). Therefore, we applied the MGBC to generate species-resolved taxonomic analyses for the compilation of 2,446 global mouse gut metagenome samples used above. The abundance and prevalence profiles of mouse gut bacterial species are available in Table S4. In line with previous reports (Lesker et al., 2020), species of the phylum Firmicutes_A represented 15 of the top 20 most prevalent species in the mouse microbiota (Figure S2A), while CAG-485 sp002362485 (recently proposed as "*Sangeribacter muris*" [Forster et al., 2021]) was the most prevalent species. As variation in the mouse gut microbiota is recognized as a confounding factor of mouse studies (Baker, 2016; Forster et al., 2021; Stappenbeck and Virgin, 2016), we assessed the effect of host genetic and environmental factors on compositional variation in the mouse gut microbiota (Figure S2B; Table S5). The study itself was the most impactful factor, explaining 40.1% of the variance in microbiota composition (Figure S2B), followed by the institute in which the study was performed (38.0%). We next assessed the microbiota of C57BL/6 "control" mice from different institutes (Figure S2C). These institutes exhibited large differences in abundance of key microbial species, including known phenotypically important bacterial species such as *Bacteroides thetaiotaomicron* and CAG-485 sp002362485. We additionally considered the differences between the microbiotas of laboratory and wild mice. Species prevalence between laboratory and wild mouse cohorts correlated strongly (Figure S2D) although species were found to be differentially abundant (Figure S2E). Taken together, these enhanced analyses using the MGBC indicate that while institutional housing environments represent a substantial source of variation and may in part explain the irreproducibility observed between mouse studies, the microbiota of laboratory mice is more similar to that of wild mice than previously suggested (Rosshart et al., 2019, 2017).

Taxonomic versus functional relationships between human and mouse gut bacteria

Gene cluster analyses have estimated that taxonomic overlap between the human and mouse gut microbiotas is below 4% (Xiao et al., 2015). Using a whole-genome approach, we compared the MGBC with the high-quality genomes of the Unified Human Gastrointestinal Genome (UHGG) collection (Almeida et al., 2021) to reveal that only 2.58% (103/3997) of species are shared between the human and mouse gut microbiotas (Figure S3A). Of these shared species, 93.2% could be assigned a species rank by GTDB-Tk, and 55.3% have cultured representatives within the MGBC (Table S6).

While very few species are shared, it has previously been estimated using gene-level functional analyses that up to 95% of gut microbial functions are shared between humans and mice (Xiao et al., 2015). To ascertain the degree of functional overlap between the human and mouse microbiotas, we created genome-linked protein catalogs from all high-quality, non-redundant genomes of the MGBC and UHGG. Using these catalogs, we assembled pangenomes for each species cluster of the MGBC and UHGG and annotated predicted protein-coding sequences using InterProScan (Jones et al., 2014) and eggNOG (Huerta-Cepas et al., 2019, 2017). In line with previous estimates, 84.5% of KEGG orthology (KO) groups and 82.1% of InterPro (IPR) protein families are shared between the gut ecosystems of the two hosts (Figure S3B). However, due to the currently incomplete functional reference databases (Thomas and Segata, 2019), only 53.2% of proteins could be assigned a KO, and notable differences were observed in the annotation efficiency between phyla (Figure S3C) as well as between host organisms (Figure S3D). Taken together, these analyses confirm

CellPress
OPEN ACCESS

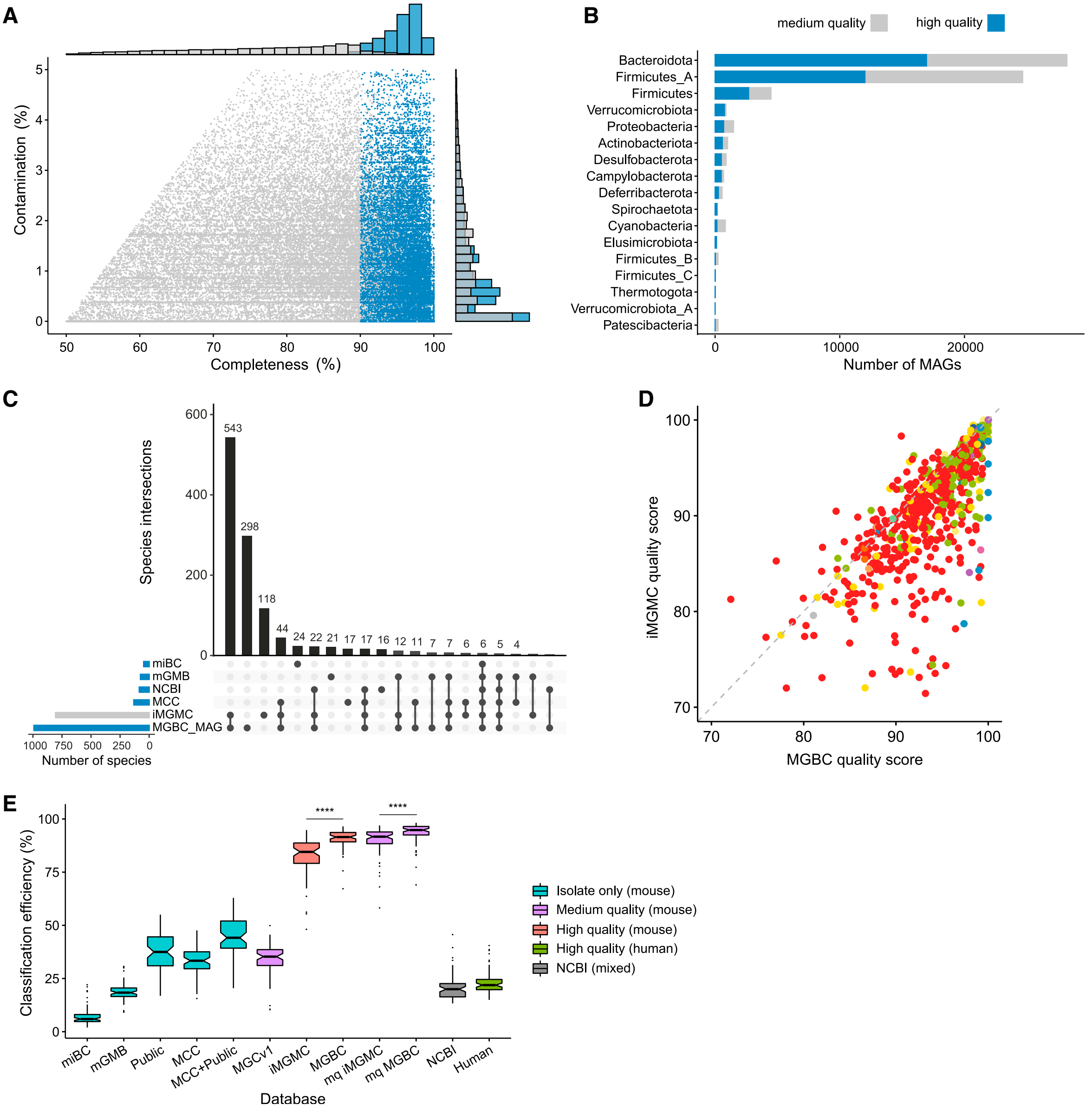

Figure 3. Genome quality evaluation and benchmarking of the MGBC

(A) Completeness and contamination of MAGs of the MGBC. Using a modified MIMAGs criteria, 26,640 MAGs were defined as high-quality (blue) ($\geq$90% completeness, $\leq$5% contamination, metrics of genome fragmentation). Quality estimates were generated using CheckM.

(B) Phylum-level distribution of high-quality and medium-plus MAGs.

(C) Upset plot illustrating the intersections of species between the contributing isolate collections and MAGs of the MGBC (blue). The iMGMC has been included for comparison (gray).

(D) Comparison of representative genome quality for shared species between the MGBC and iMGMC. Genome quality score: QS = Completeness $-$ 5 $\times$ Contamination. Color represents phylum.

(E) Read classification rates of 64 independent mouse gut metagenome samples using different custom Kraken2 databases. Box plot color indicates the origins of the genomes used to build each database. Only genomes meeting high-quality criteria were used to build databases, except where indicated (purple). miBC, n = 43; mGMB, n = 100; public (combination of all mouse gut-derived isolates from NCBI), n = 288; MCC, n = 276; MCC+public, n = 564; MGCv1, n = 239; iMGMC, n = 8,509; MGBC, n = 26,640; mq iMGMC, n = 18,306; mq MGBC, n = 65,907; NCBI (standard database), n = 97,603; human (representative genomes of the UHGG), n = 3,006. Significance was determined for selected comparisons using paired t tests, ****$p < 0.0001$.

that while functionality is largely conserved, the taxonomic locations of these shared functions likely differ between mice and humans. However, previous resources have not been sufficient to map these taxonomic locations between hosts.

To begin to resolve these limitations for translating microbiota findings between humans and mice, we next quantified functional distances between species pangenomes using a combination of functional annotation schemes (KEGG [Kanehisa

et al., 2017], MetaCyc [Caspi et al., 2016], InterPro, CAZy [Lombard et al., 2014], and GO [Ashburner et al., 2000]) and examined the functional similarities between species in the context of their taxonomic relationships. Interpangenome functional and taxonomic relationships were significantly conserved at a broad phylogenetic scale (Figure 4A). For each human bacterial species, we compared the identity of the closest mouse taxonomic species with the closest mouse functional species and found that these were only the same taxon in 47.0% of cases. We next stratified these analyses by the shared taxonomic rank of the closest mouse taxonomic species (Figure 4B). Where the closest taxonomic mouse species was assigned to the same species as the human species (i.e., shared species), the closest functional mouse species was the same taxon in 99% of cases. The only exception was human *Phocaeicola dorei* (formerly *Bacteroides dorei*), a shared species in the mouse gut microbiota, which was functionally closer to mouse *Phocaeicola vulgatus* (formerly *Bacteroides vulgatus*) than mouse *Phocaeicola dorei*. The functional pathways that underly these differences include bacterial cell wall biosynthesis, pyocyanin biosynthesis, and vitamin B12 biosynthesis pathways (Table S10). Where the closest taxonomic mouse species belonged to the same genus (e.g., human *Bifidobacterium infantis* and mouse *B. globosum*), the closest functional mouse species was only the same taxon in 57.5% of cases, dropping to 37.2% and 31.7% when the closest taxonomic mouse species was shared at the family- and order- level, respectively (Figure 4B). Functional distances increase as a function of taxonomic distance (Figure 4C), suggesting that the closest functional species at higher taxonomic ranks are not likely to be functionally identical but rather may represent the most likely species for recapitulating associated functions or phenotypes of interest. Divergent taxonomy-function relationships are not confined to any particular taxonomic clade but are present in every phylum of the gut microbiota (Figure 4D). These findings indicate that the closest taxonomic neighbor, at every taxonomic rank, is not necessarily the closest related species functionally and, therefore, might not be the best candidate for investigating microbiota functions between hosts.

Taxonomic locations of drug metabolism genes in the microbiotas of mice and humans

Global functional comparisons between species of the human and mouse gut microbiotas may serve as a starting point to translate microbial functions between hosts; however, it is likely more useful to identify functionally equivalent species between hosts at the level of an individual function or gene product. One biologically important example of this is drug metabolism by the gut microbiota, where the drug metabolizing capacity of a species can have implications for drug therapy (Collins and Patterson, 2020; Maini Rekdal et al., 2019). As mice are common models for preclinical pharmaceutical research, we leveraged the MGBC to examine the conservation status and taxonomic location of 34 experimentally validated drug metabolizing genes from the human gut microbiota (Haiser et al., 2013; Maini Rekdal et al., 2019; Ridlon et al., 2013; Zimmermann et al., 2019a, 2019b) (Table S7). For 27 genes (79.4%), the most dominant species in the human gut microbiota was shared in the mouse, and the same gene product ($\geq$95% sequence identity) was found in both human- and mouse-derived species pangenomes (Figures 5A and 5B). However, in 37% (10/27) of these cases, the most dominant species that encoded these genes in the murine host differed from the most dominant species in humans—for drug metabolizing genes described in human *Phocaeicola dorei* (formerly *Bacteroides dorei*), the most dominant gene-encoding species in the mouse microbiota is *Phocaeicola vulgatus* (formerly *Bacteroides vulgatus*) (Figure 5B). Notably, our global species-resolved functional analyses had identified mouse-derived *Phocaeicola vulgatus* as the closest functional species to human-derived *Phocaeicola dorei*, instead of the more closely, taxonomically related mouse-derived *Phocaeicola dorei*.

Where the human-derived species was not shared with the mouse gut microbiota (7/34), an equivalent gene product could not be located in the high-quality genomes of the MGBC (Figure 5C), even at 50% sequence identity. While these analyses indicate that homologous gene products are unlikely to be encoded by dominant species of the mouse gut microbiota, it is possible that homologous genes might be encoded by subdominant species that have been sequenced with insufficient coverage to generate high-quality bins. Therefore, we searched the medium-quality genomes of our collection for these genes. Although no genes with $\geq$95% sequence identity were identified, we found hits for four drug metabolizing genes with $\geq$50% identity (Table S7). While experiments using cultured isolates are necessary to validate these *in silico* predictions, the MGBC provides the species-resolved taxonomic locations of functions of interest that enable further functional and phenotypic studies.

Validating butyrate synthesis by different human and mouse microbes

Butyrate-producing species are also functionally important members of the human gut microbiota and are associated with clinical outcomes of diseases as diverse as inflammatory bowel disease (Parada Venegas et al., 2019) and depression (Caspani et al., 2019). In addition, butyrate is involved in regulating host metabolism (Donohoe et al., 2011), sleep (Szentirmai et al., 2019), and healthy cognitive functioning (Silva et al., 2020) and is important for induction of peripheral T regulatory cells (Atarashi et al., 2011; Furusawa et al., 2013). *Faecalibacterium prausnitzii*, a canonical butyrate-producing species, has been associated with improved outcomes for inflammatory bowel disease (Carlsson et al., 2013; Sokol et al., 2008), but it does not colonize well in the mouse gut (Aluthge et al., 2020; Lundberg et al., 2020), limiting the utility of mouse models to study these clinical associations. To demonstrate how the MGBC can be leveraged to potentially overcome such challenges, we identified the equivalent butyrogenic species of the mouse gut microbiota.

Butyrate is synthesized from dietary fiber or from amino acids such as glutamate and lysine, culminating in the conversion of butyryl-CoA to butyrate via either butyrate CoA-transferase (BCOAT; direct pathway) or a phosphotransferase pathway (PTB/BUK; indirect pathway) (Louis and Flint, 2017) (Figure S4A). To identify the butyrate-producing species of the mouse gut microbiota and compare the taxonomic locations of this function with the human gut ecosystem, we ranked species according to the number of genomes encoding terminal

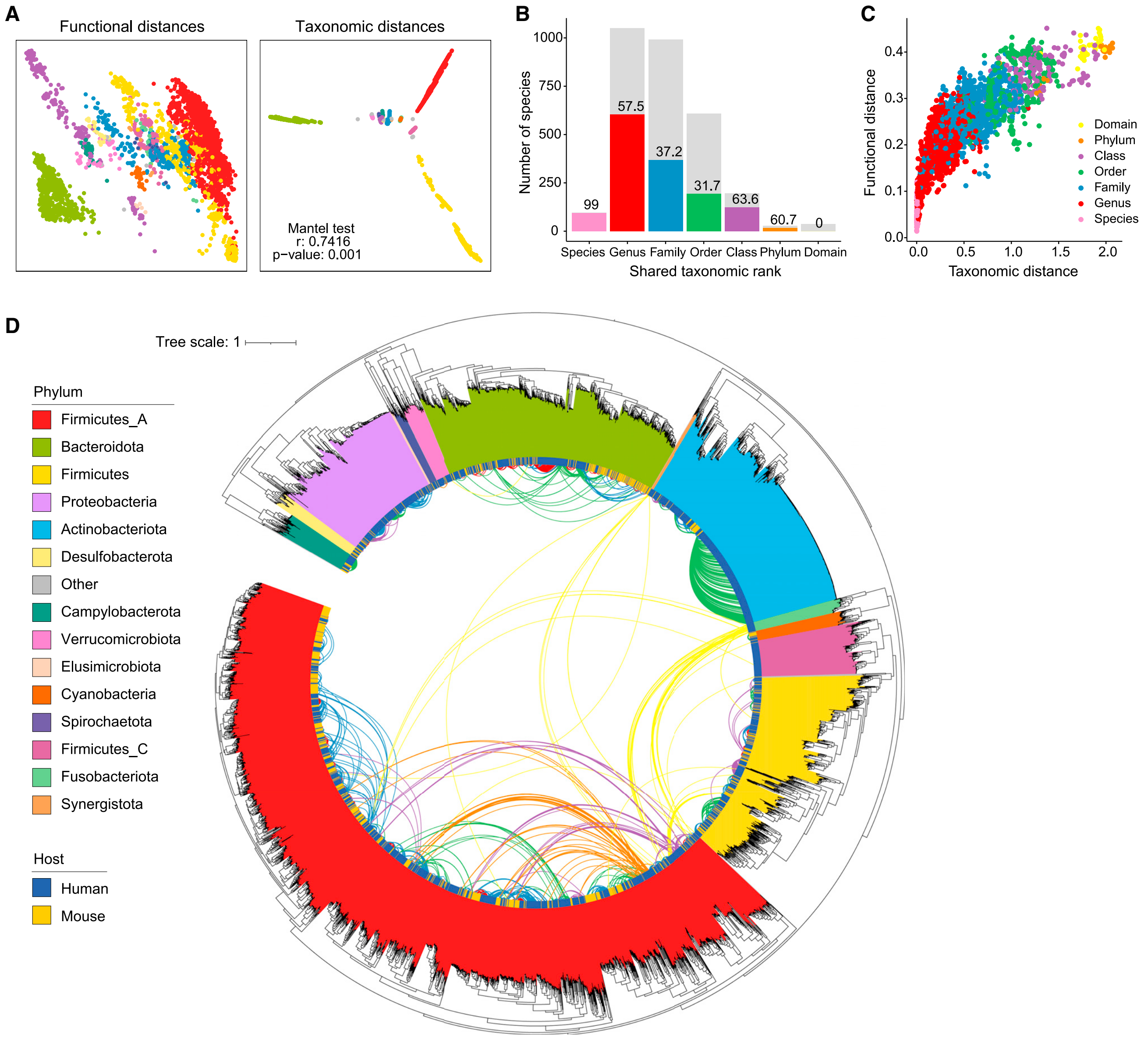

Figure 4. Taxonomy-function relationships between species of the human and mouse microbiotas

Figure360▷ For a Figure360 author presentation of this figure, see https://doi.org/10.1016/j.chom.2021.12.003.

(A) Principal coordinate analyses for functional (left) and taxonomic (right) relationships between all species of human and mouse gut microbiota. Each data point represents a single species cluster, and point color denotes phylum. Functional analyses use Jaccard distances between pangenomic functional profiles of each species. Taxonomic distances represent phylogenetic branch lengths between species calculated from alignment of 120 core genes. Distance matrices used for ordination were compared using the Mantel test (r = 0.7416, p = 0.001).

(B) Taxonomy-function relationships between human- and mouse-derived bacterial species, stratified by shared taxonomic level. Bars indicate distribution of shared taxonomic rank between closest taxonomically related species. Colored bars and bar statistics indicate number and percentage, respectively, of paired species at each rank where the closest functionally related species is the same taxon as the closest taxonomic relative.

(C) Scatterplot comparing taxonomic distance with functional distance for each human-derived species and the closest taxonomically related mouse-derived species. Color indicates the shared taxonomic rank between these species.

(D) Inverted maximum likelihood tree of the 4,100 species of the human and mouse gut microbiotas. External branches represent phylogenetic relationships between representative genome of each species. Internal connections illustrate closest functionally related species between hosts. Connections are only shown when the closest taxonomically and functionally related taxa differ. Clade color represents phylum of each species, and the inside color bar denotes the host. Color of internal connections indicates shared taxonomic rank of the closest functionally related species.

pathway genes between hosts (Figures 6A and 6B). Model butyrate producers of the human gut microbiota, including *Agathobacter rectalis* (homotypic synonym: *Eubacterium rectale*), *A. faecis* (homotypic synonym: *Roseburia faecis*), *Faecalibacterium prausnitzii*, and *Anaerostipes hadrus* featured among the top 20 most dominant BCOAT-encoding species

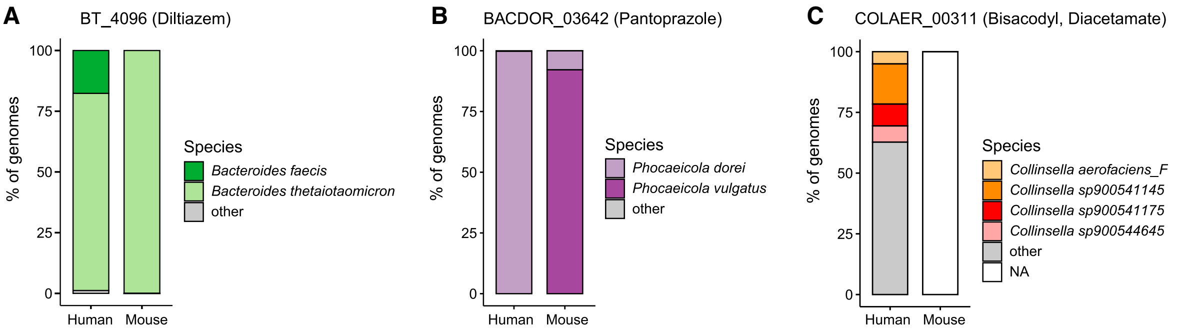

Figure 5. Taxonomic locations of drug metabolism genes between host microbiotas
(A–C) Representative examples of taxonomic locations of drug metabolism genes between host microbiotas. Data illustrate the species-level contribution of genomes encoding the indicated drug metabolism gene ($\geq$95% sequence identity). Genes and associated predicted functions are either (A) shared with a conserved taxonomic location, (B) shared with a different taxonomic location, or (C) not shared between hosts.

from the human gut (Figure 6A, top), while members of the *Coprococcus* genus were among the top 20 PTB/BUK-encoding species (Figure 6A, bottom). Importantly, all the most dominant butyrate-producing species from both host organisms were host-specific. While no human butyrate producers were previously uncharacterized as determined by GTDB, in the mouse, 17 of the top 20 BCOAT-encoding species (85%) and 11 of the top 20 PTB/BUK-encoding species (55%) could not be assigned to a species-level taxonomy (Figure 6B).

As 94.2% of predicted butyrate-producing species belong to the Firmicutes_A phylum, we considered the taxonomic locations within this phylum of the butyrate terminal pathway genes between hosts (Figure 6C). Both pathways are largely conserved within the same taxonomic clades between hosts (Figure 6C); however, the most dominant species in each host for each pathway do not represent the closest species phylogenetically, suggesting that there are likely to be host-specific niche factors affecting species dominance.

Cultured isolates are essential for validating *in silico* functional predictions from genomic data. Utilizing our MCC, we identified isolates that were predicted to produce butyrate via each terminal pathway according to genomic functional annotations. We selected three isolates for each pathway that ranked in the top 30 most abundant predicted butyrate-producing species (Figure S4B) based on the species abundance profiles generated from 2,446 mouse gut metagenomic samples (Table S2). We then tested the capacity of these isolates to synthesize butyrate in broth monoculture. Isolates for known butyrate-producing species from the human gut microbiota, *Coprococcus eutactus* and *Eubacterium rectale* (Sorbara et al., 2020), were included as positive controls, and an isolate from the MCC that lacked predicted butyrate terminal pathway genes was included as a negative control. All predicted isolates produced butyrate (Figure 6D; Table S9), although *Kineothrix sp000403275* grew poorly in broth culture (OD < 0.1) and, therefore, exhibited only a low-level increase in butyrate compared with the negative control isolate *Lachnospiraceae_NOV MGBC000113*. Together, these findings demonstrate that the MGBC and MCC resources we have generated enable identification and experimental investigation of functionally equivalent gut bacterial species between hosts.

DISCUSSION

In this study, we generated the MGBC as a resource for translating microbiota findings between the host-specific microbiotas of humans and mice by providing access to the taxonomic locations of functions of interest in the gut microbiotas of both hosts. We demonstrated the utility of our resource with butyrate synthesis and drug metabolism as applied examples; however, these represent just two of many biologically and medically important functions of the gut microbiota. For example, bile acid metabolism is largely divergent between human and mouse gut microbiotas and has been shown to play a key role in susceptibility to enteric infection (Buffie et al., 2015). The ability to further explore this metabolic pathway in mouse models could yield important mechanistic insights for targeted therapeutics. The MGBC is therefore accompanied by a toolkit (https://github.com/BenBeresfordJones/MGBC-Toolkit) to allow users to query any function of interest. It provides users the ability to look for functions via annotation ID (KEGG, InterPro, COG, eggNOG, and GO) and the ability to screen for gene products at the sequence level, facilitating identification of nonannotated genes and predicted functions of interest. Our bacterial catalog also enables improved correlative analyses of mouse gut metagenomes through increased coverage of the mouse gut microbiome and expands the study of causation in the mouse gut microbiota through increased availability of cultured isolates. Our toolkit and analyses improve our understanding of the taxonomy-function relationships of bacterial species from the microbiotas of different hosts and reveal that the closest taxonomically related species is not necessarily the most functionally equivalent.

Understanding the functional potential encoded within microbial genomes is key to understanding the implications of taxonomically divergent microbiotas between host organisms (Thomas and Segata, 2019). Our results, as well as those of other studies (Almeida et al., 2021), indicate that over 40% of predicted proteins have no representation in functional reference databases. These functional unknowns limit the resolution of interhost functional analyses and remain one of the main limiting factors for the microbiome field at large (Thomas and Segata, 2019). In order to partly mitigate these limitations and using

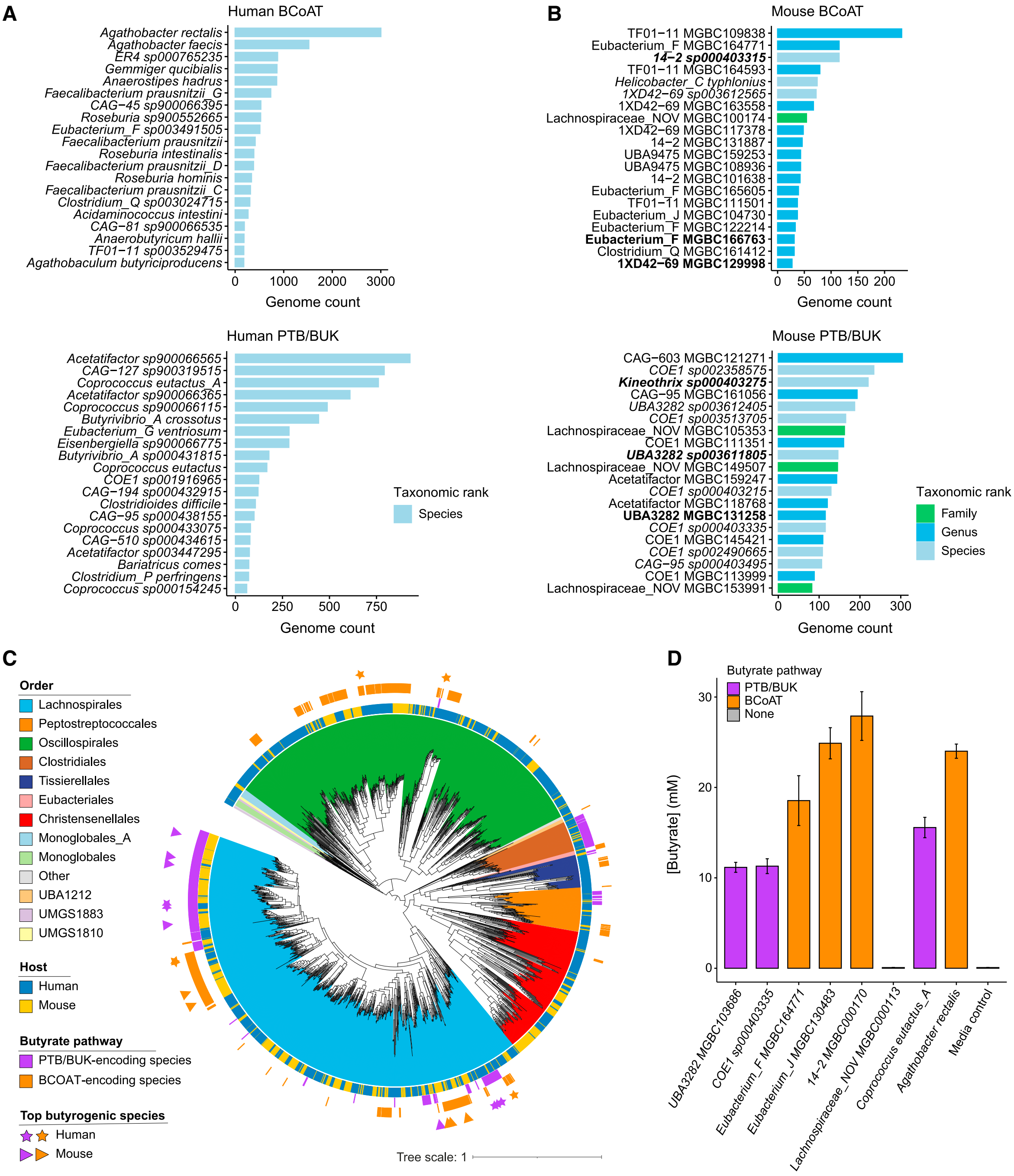

Figure 6. Identification and validation of butyrate-producing species between hosts

(A and B) The most dominant butyrate-producing species of the human (A) and mouse (B) gut microbiotas, utilizing either the BCOAT (top) or PTB/BUK (bottom) pathways. Color indicates the lowest assigned taxonomic rank for each species by GTDB-Tk, either known species (light blue), novel species (dark blue), or novel genera (green).

(C) Maximum likelihood tree of the representative genomes for species of the Firmicutes_A phylum. Color range represents the order-level taxonomy, and the innermost color bar denotes the host organism. The outer color bars indicate predicted butyrate-producing species using the BCOAT pathway (purple) or the PTB-BUK pathway (orange). The top 5 most dominant butyrogenic pathway encoding species for each host are marked with a colored triangle (mouse) or star (human).

(D) Butyrate production by bacterial isolates in broth monoculture. Bar color indicates the encoded pathway for butyrate synthesis.

drug metabolism genes as examples, we used taxonomically contextualized protein catalogs to perform sequence-level interhost functional comparisons for nonannotated functions. However, this approach does not enable prediction of functional capacity from genomic data. Future studies combining genome-level data with experimental functional characterization, enabled by large-scale genome and isolate catalogs (Sorbara et al., 2020), will therefore be required to uncover this unexplored functionality. Notably, nearly 77% of species represented in the MGBC lack a cultured representative, hindering experimental validation of associated phenotypes and functions. Genomic insights can be used to facilitate targeted culturing techniques and improve the representation of cultured species (Browne et al., 2016; Cross et al., 2019). Future studies that increase cultured diversity will also be highly valuable to advancing the therapeutic potential of the microbiota field.

Highly complete genomes with low levels of contamination are required for accurate functional analyses, necessitating the use of stringent quality control thresholds and validated binning methods when curating genome catalogs. In addition, a consistent level of genome quality is required to combine both isolate genomes and MAGs in analyses (Almeida et al., 2021). However, as lower quality genomes, often representing minor or rare species of the gut microbiota (Pedron et al., 2019), are excluded from these analyses, there is a loss of covered taxonomic and functional diversity, which is then inaccessible for comparison. While minor species of the gut microbiota may not be sequenced with sufficient coverage to facilitate complete genome binning, future microbiome studies implementing long-read or deeper sequencing and improved binning methods will be useful for expanding the known taxonomic and functional diversity of the mouse gut microbiota. In addition, standard tools for estimating genome completeness exhibit bias against certain taxonomic clades and represent technical challenges in the compilation of fully comprehensive genome collections. For example, genomes of the large clade of uncultured bacteria, candidate phyla radiation (CPR), cannot be assigned a high-quality genome status using current single copy number gene methods (Brown et al., 2015). Studies aiming to address these technical limitations, such as the recently published Genome UNClutterer (GUNC) tool (Orakov et al., 2021), will drive further improvements to the comprehensiveness and quality of genome catalogs.

Bacteria represent the most dominant members of the mouse gut ecosystem, contributing up to 90% of the genetic material in metagenomic samples according to our analyses; however, other domains including fungi, microeukaryotes, and viruses remain understudied in the context of host-specific microbiotas. It is likely that these biomes will contribute additional functional and phenotypic complexities to modeling human disease in mice, and further study of these domains may yield additional avenues for clinical intervention. Targeted microbiota therapies, live bacterial therapeutics, rationally designed drugs, and microbial biomarkers of disease are just some of the clinical developments that microbiota research aims to deliver to improve outcomes of human health and disease. Mouse models will continue to play a central role in this research, and therefore, the isolate, genome, and protein catalogs presented in our study represent important developments in understanding and tackling the obstacles posed by host-specific microbiota and provide a starting point toward efficient and informed translation of gut microbiota research between humans and mice.

STAR★METHOD

Detailed methods are provided in the online version of this paper and include the following:

- KEY RESOURCES TABLE
- RESOURCE AVAILABILITY
  - Lead contact
  - Materials availability
  - Data and code availability
- EXPERIMENTAL MODEL AND SUBJECT DETAILS
  - Faecal samples from mice
  - Bacterial culture
- METHOD DETAILS
  - Curation of public mouse gut-derived isolate genomes
  - Curation of public human gut-derived bacterial genomes
  - Mouse gut metagenome cohort curation
  - Whole-genome sequencing and assembly
  - Faecal sample collection and shotgun metagenomic sequencing
  - Workflow for metagenome-assembled genome (MAG) synthesis
  - Quality control of genome assemblies
  - Taxonomic classification of genomes and species clustering
  - Determining prior cultured status of isolates
  - Benchmarking binner performance for production of high-quality MAGs
  - Comparison to other mouse microbiota resources
  - Construction of phylogenetic trees
  - Metagenome classification and analysis
  - Taxonomic comparison of the mouse and human gut microbiotas
  - Pangenome synthesis and functional annotation
  - Taxonomic and functional distance analyses
  - Functional comparison of *Phocaeicola* species
  - Drug metabolism analyses
  - Butyrate synthesis analyses
  - Validating butyrate terminal pathway predictions using isolate cultures
  - Short chain fatty acid quantitation by GC-MS
- QUANTIFICATION AND STATISTICAL ANALYSIS

SUPPLEMENTAL INFORMATION

Supplemental information can be found online at https://doi.org/10.1016/j.chom.2021.12.003.

AUTHOR CONTRIBUTIONS

B.S.B.-J., T.D.L., and V.A.P. conceived the study. S.C.F. initiated culturing of isolates. M.D.S., G.N., E.V., H.P.B., and B.S.B.-J. cultured isolates. B.S.B.-J. curated genomes and metagenomes, generated MAGs, performed computational analyses, and analyzed and assembled data. D.J.B. and J.R.C. performed and analyzed metabolomics. A.T.S. and B.S.B.-J. provided metagenomes from Wellcome Sanger Institute. N.K., K.V., and A.A. advised on

computational analyses. A.A. and B.S.B.-J. wrote code for computational analyses. B.S.B.-J., T.D.L., and V.A.P. wrote the article.

ACKNOWLEDGMENTS

V.A.P. is supported by Sir Henry Dale Fellowship jointly funded by the Wellcome Trust and the Royal Society (206245/Z/17/Z). B.S.B.-J. is supported by a studentship from the Rosetrees Trust (A2194). T.D.L. is supported by the Wellcome Trust (098051). We thank Rahul Roychoudhuri and Paula Kuo from the Babraham Institute for providing mouse feces for metagenomic sequencing.

DECLARATION OF INTERESTS

T.D.L. is a founder and CSO of Microbiotica. The other authors declare no competinginterests.

Received: August 5, 2021
Revised: October 5, 2021
Accepted: November 30, 2021
Published: December 30, 2021

REFERENCES

Aitchison, J. (1992). On criteria for measures of compositional difference. Math. Geol. *24*, 365–379. https://doi.org/10.1007/BF00891269.

Almeida, A., Mitchell, A.L., Boland, M., Forster, S.C., Gloor, G.B., Tarkowska, A., Lawley, T.D., and Finn, R.D. (2019). A new genomic blueprint of the human gut microbiota. Nature *568*, 499–504. https://doi.org/10.1038/s41586-019-0965-1.

Almeida, A., Nayfach, S., Boland, M., Strozzi, F., Beracochea, M., Shi, Z.J., Pollard, K.S., Sakharova, E., Parks, D.H., Hugenholtz, P., et al. (2021). A unified catalog of 204,938 reference genomes from the human gut microbiome. Nat. Biotechnol. *39*, 105–114. https://doi.org/10.1038/s41587-020-0603-3.

Alneberg, J., Bjarnason, B.S., de Bruijn, I., Schirmer, M., Quick, J., Ijaz, U.Z., Lahti, L., Loman, N.J., Andersson, A.F., and Quince, C. (2014). Binning metagenomic contigs by coverage and composition. Nat. Methods *11*, 1144–1146. https://doi.org/10.1038/nmeth.3103.

Aluthge, N.D., Tom, W.A., Bartenslager, A.C., Burkey, T.E., Miller, P.S., Heath, K.D., Kreikemeier-Bower, C., Kittana, H., Schmaltz, R.J., Ramer-Tait, A.E., and Fernando, S.C. (2020). Differential longitudinal establishment of human fecal bacterial communities in germ-free porcine and murine models. Commun. Biol. *3*, 760. https://doi.org/10.1038/s42003-020-01477-0.

Armour, C.R., Nayfach, S., Pollard, K.S., and Sharpton, T.J. (2019). A metagenomic meta-analysis reveals functional signatures of health and disease in the human gut microbiome. mSystems *4*. e00332–e18. https://doi.org/10.1128/mSystems.00332-18.

Ashburner, M., Ball, C.A., Blake, J.A., Botstein, D., Butler, H., Cherry, J.M., Davis, A.P., Dolinski, K., Dwight, S.S., Eppig, J.T., et al. (2000). Gene ontology: tool for the unification of biology. The Gene Ontology Consortium. Nat. Genet. *25*, 25–29. https://doi.org/10.1038/75556.

Atarashi, K., Tanoue, T., Shima, T., Imaoka, A., Kuwahara, T., Momose, Y., Cheng, G., Yamasaki, S., Saito, T., Ohba, Y., et al. (2011). Induction of colonic regulatory T cells by indigenous Clostridium species. Science *331*, 337–341. https://doi.org/10.1126/science.1198469.

Baker, M. (2016). 1,500 Scientists lift the lid on reproducibility. Nature *533*, 452–454. https://doi.org/10.1038/533452a.

Boetzer, M., Henkel, C.V., Jansen, H.J., Butler, D., and Pirovano, W. (2011). Scaffolding pre-assembled contigs using SSPACE. Bioinformatics *27*, 578–579. https://doi.org/10.1093/bioinformatics/btq683.

Boetzer, M., and Pirovano, W. (2012). Toward almost closed genomes with GapFiller. Genome Biol *13*, R56. https://doi.org/10.1186/gb-2012-13-6-r56.

Bowers, R.M., Kyrpides, N.C., Stepanauskas, R., Harmon-Smith, M., Doud, D., Reddy, T.B.K., Schulz, F., Jarett, J., Rivers, A.R., Eloe-Fadrosh, E.A., et al. (2017). Minimum information about a single amplified genome (MISAG) and a metagenome-assembled genome (MIMAG) of bacteria and archaea. Nat. Biotechnol. *35*, 725–731. https://doi.org/10.1038/nbt.3893.

Brown, C.T., Hug, L.A., Thomas, B.C., Sharon, I., Castelle, C.J., Singh, A., Wilkins, M.J., Wrighton, K.C., Williams, K.H., and Banfield, J.F. (2015). Unusual biology across a group comprising more than 15% of domain Bacteria. Nature *523*, 208–211. https://doi.org/10.1038/nature14486.

Browne, H.P., Forster, S.C., Anonye, B.O., Kumar, N., Neville, B.A., Stares, M.D., Goulding, D., and Lawley, T.D. (2016). Culturing of ‘unculturable’ human microbiota reveals novel taxa and extensive sporulation. Nature *533*, 543–546. https://doi.org/10.1038/nature17645.

Buffie, C.G., Bucci, V., Stein, R.R., McKenney, P.T., Ling, L., Gobourne, A., Liu, H., Kinnebrew, M., Viale, A., et al. (2015). Precision microbiome reconstitution restores bile acid mediated resistance to Clostridium difficile. Nature *517*, 205–208. https://doi.org/10.1038/nature13828.

Camacho, C., Coulouris, G., Avagyan, V., Ma, N., Papadopoulos, J., Bealer, K., and Madden, T.L. (2009). Blast+: architecture and applications. BMC Bioinformatics *10*, 421. https://doi.org/10.1186/1471-2105-10-421.

Carlsson, A.H., Yakymenko, O., Olivier, I., Håkansson, F., Postma, E., Keita, A.V., and Söderholm, J.D. (2013). Faecalibacterium prausnitzii supernatant improves intestinal barrier function in mice DSS colitis. Scand. J. Gastroenterol. *48*, 1136–1144. https://doi.org/10.3109/00365521.2013.828773.

Caspani, G., Kennedy, S., Foster, J.A., and Swann, J. (2019). Gut microbial metabolites in depression: understanding the biochemical mechanisms. Microb. Cell *6*, 454–481. https://doi.org/10.15698/mic2019.10.693.

Caspi, R., Billington, R., Ferrer, L., Foerster, H., Fulcher, C.A., Keseler, I.M., Kothari, A., Krummenacker, M., Latendresse, M., Mueller, L.A., et al. (2016). The MetaCyc database of metabolic pathways and enzymes and the BioCyc collection of pathway/genome databases. Nucleic Acids Res *44*, D471–D480. https://doi.org/10.1093/nar/gkv1164.

Chaumeil, P.A., Mussig, A.J., Hugenholtz, P., and Parks, D.H. (2019). GTDB-Tk: a toolkit to classify genomes with the Genome Taxonomy Database. Bioinformatics *36*, 1925–1927. https://doi.org/10.1093/bioinformatics/btz848.

Chen, R.Y., Mostafa, I., Hibberd, M.C., Das, S., Mahfuz, M., Naila, N.N., Islam, M.M., Huq, S., Alam, M.A., Zaman, M.U., et al. (2021). A microbiota-directed food intervention for undernourished children. N. Engl. J. Med. *384*, 1517–1528. https://doi.org/10.1056/NEJMoa2023294.

Chung, H., Pamp, S.J., Hill, J.A., Surana, N.K., Edelman, S.M., Troy, E.B., Reading, N.C., Villablanca, E.J., Wang, S., Mora, J.R., et al. (2012). Gut immune maturation depends on colonization with a host-specific microbiota. Cell *149*, 1578–1593. https://doi.org/10.1016/j.cell.2012.04.037.

Collins, S.L., and Patterson, A.D. (2020). The gut microbiome: an orchestrator of xenobiotic metabolism. Acta Pharm. Sin. B *10*, 19–32. https://doi.org/10.1016/j.apsb.2019.12.001.

Cross, K.L., Campbell, J.H., Balachandran, M., Campbell, A.G., Cooper, C.J., Griffen, A., Heaton, M., Joshi, S., Klingeman, D., Leys, E., et al. (2019). Targeted isolation and cultivation of uncultivated bacteria by reverse genomics. Nat. Biotechnol. *37*, 1314–1321. https://doi.org/10.1038/s41587-019-0260-6.

Cryan, J.F., O’Riordan, K.J., Sandhu, K., Peterson, V., and Dinan, T.G. (2020). The gut microbiome in neurological disorders. Lancet Neurol *19*, 179–194. https://doi.org/10.1016/S1474-4422(19)30356-4.

Donohoe, D.R., Garge, N., Zhang, X., Sun, W., O’Connell, T.M., Bunger, M.K., and Bultman, S.J. (2011). The microbiome and butyrate regulate energy metabolism and autophagy in the mammalian colon. Cell Metab *13*, 517–526. https://doi.org/10.1016/j.cmet.2011.02.018.

Duncan, S.H., Hold, G.L., Harmsen, H.J.M., Stewart, C.S., and Flint, H.J. (2002). Growth requirements and fermentation products of Fusobacterium prausnitzii, and a proposal to reclassify it as Faecalibacterium prausnitzii gen. nov., comb. nov. Int. J. Syst. Evol. Microbiol. *52*, 2141–2146. https://doi.org/10.1099/00207713-52-6-2141.

Forster, S.C., Clare, S., Beresford-Jones, B.S., Harcourt, K., Notley, G., Stares, M., Kumar, N., Soderholm, A.T., Adoum, A., Wong, H., et al. (2021). Novel gut pathobionts confound results in a widely used mouse model of

human inflammatory disease. bioRxiv. https://doi.org/10.1101/2021.02.09.430393.

Forster, S.C., Kumar, N., Anonye, B.O., Almeida, A., Viciani, E., Stares, M.D., Dunn, M., Mkandawire, T.T., Zhu, A., Shao, Y., et al. (2019). A human gut bacterial genome and culture collection for improved metagenomic analyses. Nat. Biotechnol. *37*, 186–192. https://doi.org/10.1038/s41587-018-0009-7.

Furusawa, Y., Obata, Y., Fukuda, S., Endo, T.A., Nakato, G., Takahashi, D., Nakanishi, Y., Uetake, C., Kato, K., Kato, T., et al. (2013). Commensal microbe-derived butyrate induces the differentiation of colonic regulatory T cells. Nature *504*, 446–450. https://doi.org/10.1038/nature12721.

Haiser, H.J., Gootenberg, D.B., Chatman, K., Sirasani, G., Balskus, E.P., and Turnbaugh, P.J. (2013). Predicting and manipulating cardiac drug inactivation by the human gut bacterium Eggerthella lenta. Science *341*, 295–298. https://doi.org/10.1126/science.1235872.

Harrison, P.W., Ahamed, A., Aslam, R., Alako, B.T.F., Burgin, J., Buso, N., Courtot, M., Fan, J., Gupta, D., Haseeb, M., et al. (2021). The European Nucleotide Archive in 2020. Nucleic Acids Res *49*, D82–D85. https://doi.org/10.1093/nar/gkaa1028.

Huerta-Cepas, J., Forslund, K., Coelho, L.P., Szklarczyk, D., Jensen, L.J., von Mering, C., and Bork, P. (2017). Fast genome-wide functional annotation through orthology assignment by eggNOG-mapper. Mol. Biol. Evol. *34*, 2115–2122. https://doi.org/10.1093/molbev/msx148.

Huerta-Cepas, J., Szklarczyk, D., Heller, D., Hernández-Plaza, A., Forslund, S.K., Cook, H., Mende, D.R., Letunic, I., Rattei, T., Jensen, L.J., et al. (2019). eggNOG 5.0: a hierarchical, functionally and phylogenetically annotated orthology resource based on 5090 organisms and 2502 viruses. Nucleic Acids Res *47*, D309–D314. https://doi.org/10.1093/nar/gky1085.

Jain, C., Rodriguez-R, L.M., Phillippy, A.M., Konstantinidis, K.T., and Aluru, S. (2018). High throughput ANI analysis of 90K prokaryotic genomes reveals clear species boundaries. Nat. Commun. *9*, 5114. https://doi.org/10.1038/s41467-018-07641-9.

Jassal, B., Matthews, L., Viteri, G., Gong, C., Lorente, P., Fabregat, A., Sidiropoulos, K., Cook, J., Gillespie, M., Haw, R., et al. (2020). The reactome pathway knowledgebase. Nucleic Acids Res *48*, D498–D503. https://doi.org/10.1093/nar/gkz1031.

Johnson, M., Zaretskaya, I., Raytselis, Y., Merezhuk, Y., McGinnis, S., and Madden, T.L. (2008). NCBI BLAST: a better web interface. Nucleic Acids Res *36*, W5–W9. https://doi.org/10.1093/nar/gkn201.

Jones, P., Binns, D., Chang, H.Y., Fraser, M., Li, W., McAnulla, C., McWilliam, H., Maslen, J., Mitchell, A., Nuka, G., et al. (2014). InterProScan 5: genome-scale protein function classification. Bioinformatics *30*, 1236–1240. https://doi.org/10.1093/bioinformatics/btu031.

Kalyaanamoorthy, S., Minh, B.Q., Wong, T.K.F., von Haeseler, A., and Jermiin, L.S. (2017). ModelFinder: fast model selection for accurate phylogenetic estimates. Nat. Methods *14*, 587–589. https://doi.org/10.1038/nmeth.4285.

Kanehisa, M., Furumichi, M., Tanabe, M., Sato, Y., and Morishima, K. (2017). KEGG: new perspectives on genomes, pathways, diseases and drugs. Nucleic Acids Res *45*, D353–D361. https://doi.org/10.1093/nar/gkw1092.

Kang, D.D., Li, F., Kirton, E., Thomas, A., Egan, R., An, H., and Wang, Z. (2019). MetaBAT 2: an adaptive binning algorithm for robust and efficient genome reconstruction from metagenome assemblies. PeerJ *7*, e7359. https://doi.org/10.7717/peerj.7359.

Kim, C.Y., Lee, M., Yang, S., Kim, K., Yong, D., Kim, H.R., and Lee, I. (2020). Human reference gut microbiome comprising 5,414 prokaryotic species, including newly assembled genomes from under-represented Asian metagenomes. bioRxiv. https://doi.org/10.1101/2020.11.09.375873.

Lagier, J.C., Khelaifia, S., Alou, M.T., Ndongo, S., Dione, N., Hugon, P., Caputo, A., Cadoret, F., Traore, S.I., Seck, E.H., et al. (2016). Culture of previously uncultured members of the human gut microbiota by culturomics. Nat. Microbiol. *1*, 16203. https://doi.org/10.1038/nmicrobiol.2016.203.

Lagkouvardos, I., Lesker, T.R., Hitch, T.C.A., Gálvez, E.J.C., Smit, N., Neuhaus, K., Wang, J., Baines, J.F., Abt, B., Stecher, B., et al. (2019). Sequence and cultivation study of Muribaculaceae reveals novel species, host preference, and functional potential of this yet undescribed family. Microbiome *7*, 28. https://doi.org/10.1186/s40168-019-0637-2.

Lagkouvardos, I., Pukall, R., Abt, B., Foesel, B.U., Meier-Kolthoff, J.P., Kumar, N., Bresciani, A., Martínez, I., Just, S., Ziegler, C., et al. (2016). The Mouse Intestinal Bacterial Collection (miBC) provides host-specific insight into cultured diversity and functional potential of the gut microbiota. Nat. Microbiol. *1*, 16131. https://doi.org/10.1038/nmicrobiol.2016.131.

Langmead, B., Wilks, C., Antonescu, V., and Charles, R. (2019). Scaling read aligners to hundreds of threads on general-purpose processors. Bioinformatics *35*, 421–432. https://doi.org/10.1093/bioinformatics/bty648.

Lesker, T.R., Durairaj, A.C., Gálvez, E.J.C., Lagkouvardos, I., Baines, J.F., Clavel, T., Sczyrba, A., McHardy, A.C., and Strowig, T. (2020). An integrated metagenome catalog reveals new insights into the murine gut microbiome. Cell Rep *30*, 2909–2922.e6. https://doi.org/10.1016/j.celrep.2020.02.036.

Letunic, I., and Bork, P. (2021). Interactive Tree Of Life (iTOL) v5: an online tool for phylogenetic tree display and annotation. Nucleic Acids Res *49*, W293–W296. https://doi.org/10.1093/nar/gkab301.

Li, B., Selmi, C., Tang, R., Gershwin, M.E., and Ma, X. (2018). The microbiome and autoimmunity: a paradigm from the gut–liver axis. Cell. Mol. Immunol. *15*, 595–609. https://doi.org/10.1038/cmi.2018.7.

Li, D., Luo, R., Liu, C.M., Leung, C.M., Ting, H.F., Sadakane, K., Yamashita, H., and Lam, T.W. (2016). MEGAHIT v1.0: A fast and scalable metagenome assembler driven by advanced methodologies and community practices. Methods *102*, 3–11. https://doi.org/10.1016/j.ymeth.2016.02.020.

Li, J., Jia, H., Cai, X., Zhong, H., Feng, Q., Sunagawa, S., Arumugam, M., Kultima, J.R., Prifti, E., Nielsen, T., et al. (2014). An integrated catalog of reference genes in the human gut microbiome. Nat. Biotechnol. *32*, 834–841. https://doi.org/10.1038/nbt.2942.

Libertucci, J., and Young, V.B. (2019). The role of the microbiota in infectious diseases. Nat. Microbiol. *4*, 35–45. https://doi.org/10.1038/s41564-018-0278-4.

Liu, C., Zhou, N., Du, M.X., Sun, Y.T., Wang, K., Wang, Y.J., Li, D.H., Yu, H.Y., Song, Y., Bai, B.B., et al. (2020). The Mouse Gut Microbial Biobank expands the coverage of cultured bacteria. Nat. Commun. *11*, 79. https://doi.org/10.1038/s41467-019-13836-5.

Lombard, V., Golaconda Ramulu, H., Drula, E., Coutinho, P.M., and Henrissat, B. (2014). The carbohydrate-active enzymes database (CAZy) in 2013. Nucleic Acids Res *42*, D490–D495. https://doi.org/10.1093/nar/gkt1178.

Louis, P., and Flint, H.J. (2017). Formation of propionate and butyrate by the human colonic microbiota. Environ. Microbiol. *19*, 29–41. https://doi.org/10.1111/1462-2920.13589.

Lu, J., Breitwieser, F.P., Thielen, P., and Salzberg, S.L. (2017). Bracken: estimating species abundance in metagenomics data. PeerJ Comput. Sci. *3*, e104. https://doi.org/10.7717/peerj-cs.104.

Lundberg, R., Toft, M.F., Metzdorff, S.B., Hansen, C.H.F., Licht, T.R., Bahl, M.I., and Hansen, A.K. (2020). Human microbiota-transplanted C57BL/6 mice and offspring display reduced establishment of key bacteria and reduced immune stimulation compared to mouse microbiota-transplantation. Sci. Rep. *10*, 7805. https://doi.org/10.1038/s41598-020-64703-z.

Maini Rekdal, V.M., Bess, E.N., Bisanz, J.E., Turnbaugh, P.J., and Balskus, E.P. (2019). Discovery and inhibition of an interspecies gut bacterial pathway for levodopa metabolism. Science *364*, eaau6323. https://doi.org/10.1126/science.aau6323.

Martín-Fernández, J.-A., Hron, K., Templ, M., Filzmoser, P., and Palarea-Albaladejo, J. (2015). Bayesian-multiplicative treatment of count zeros in compositional data sets. Stat. Modell. *15*, 134–158. https://doi.org/10.1177/1471082X14535524.

Meziti, A., Rodriguez-R, L.M., Hatt, J.K., Peña-Gonzalez, A., Levy, K., and Konstantinidis, K.T. (2021). The reliability of metagenome-assembled genomes (MAGs) in representing natural populations: insights from comparing MAGs against isolate genomes derived from the same fecal sample. Appl. Environ. Microbiol. *87*, e02593–e02620. https://doi.org/10.1128/AEM.02593-20.

CellPress
OPEN ACCESS

Nayfach, S., Shi, Z.J., Seshadri, R., Pollard, K.S., and Kyrpides, N.C. (2019). New insights from uncultivated genomes of the global human gut microbiome. Nature *568*, 505–510. https://doi.org/10.1038/s41586-019-1058-x.

Neville, B.A., Forster, S.C., and Lawley, T.D. (2018). Commensal Koch's postulates: establishing causation in human microbiota research. Curr. Opin. Microbiol. *42*, 47–52. https://doi.org/10.1016/j.mib.2017.10.001.

Nguyen, L.T., Schmidt, H.A., von Haeseler, A., and Minh, B.Q. (2015). IQ-TREE: A fast and effective stochastic algorithm for estimating maximum-likelihood phylogenies. Mol. Biol. Evol. *32*, 268–274. https://doi.org/10.1093/molbev/msu300.

Nielsen, H.B., Almeida, M., Juncker, A., Sierakowska, R., Li, J., Sunagawa, S., Plichta, D.R., Gautier, L., Pedersen, A.G., Le Chatelier, E., et al.; MetaHIT Consortium (2014). Identification and assembly of genomes and genetic elements in complex metagenomic samples without using reference genomes. Nat. Biotechnol. *32*, 822–828. https://doi.org/10.1038/nbt.2939.

Nurk, S., Meleshko, D., Korobeynikov, A., and Pevzner, P.A. (2017). metaSPAdes: a new versatile metagenomic assembler. Genome Res *27*, 824–834. https://doi.org/10.1101/gr.213959.116.

O'Leary, N.A., Wright, M.W., Brister, J.R., Ciufo, S., Haddad, D., McVeigh, R., Rajput, B., Robbertse, B., Smith-White, B., Ako-Adjei, D., et al. (2016). Reference sequence (RefSeq) database at NCBI: current status, taxonomic expansion, and functional annotation. Nucleic Acids Res *44*, D733–D745. https://doi.org/10.1093/nar/gkv1189.

Oksanen, J., Blanchet, G., Friendly, M., Kindt, R., Legendre, P., McGlinn, D., Minchin, P.R., O'Hara, R., Simpson, G.L., Solymos, P., et al. (2014). Vegan: community ecology package, R package version 2.2-0. http://CRAN.Rproject.org/package=vegan.

Olm, M.R., Brown, C.T., Brooks, B., and Banfield, J.F. (2017). dRep: a tool for fast and accurate genomic comparisons that enables improved genome recovery from metagenomes through de-replication. ISME J *11*, 2864–2868. https://doi.org/10.1038/ismej.2017.126.

Ondov, B.D., Treangen, T.J., Melsted, P., Mallonee, A.B., Bergman, N.H., Koren, S., and Phillippy, A.M. (2016). Mash: fast genome and metagenome distance estimation using MinHash. Genome Biol *17*, 132. https://doi.org/10.1186/s13059-016-0997-x.

Orakov, A., Fullam, A., Coelho, L.P., Khedkar, S., Szklarczyk, D., Mende, D.R., Schmidt, T.S.B., and Bork, P. (2021). GUNC: detection of chimerism and contamination in prokaryotic genomes. Genome Biol *22*, 178. https://doi.org/10.1186/s13059-021-02393-0.

Page, A.J., De Silva, N., Hunt, M., Quail, M.A., Parkhill, J., Harris, S.R., Otto, T.D., and Keane, J.A. (2016). Robust high-throughput prokaryote de novo assembly and improvement pipeline for Illumina data. Microb. Genomics *2*, e000083. https://doi.org/10.1099/mgen.0.000083.

Palarea-Albaladejo, J., and Martín-Fernández, J.A. (2015). zCompositions — R package for multivariate imputation of left-censored data under a compositional approach. Chemom. Intell. Lab. Syst. *143*, 85–96. https://doi.org/10.1016/j.chemolab.2015.02.019.

Parada Venegas, D., De la Fuente, M.K., Landskron, G., González, M.J., Quera, R., Dijkstra, G., Harmsen, H.J.M., Faber, K.N., and Hermoso, M.A. (2019). Short chain fatty acids (SCFAs)-mediated gut epithelial and immune regulation and its relevance for inflammatory bowel diseases. Front. Immunol. *10*, 277. https://doi.org/10.3389/fimmu.2019.00277.

Paradis, E., and Schliep, K. (2019). ape 5.0: an environment for modern phylogenetics and evolutionary analyses in R. Bioinformatics *35*, 526–528. https://doi.org/10.1093/bioinformatics/bty633.

Park, J.C., and Im, S.H. (2020). Of men in mice: the development and application of a humanized gnotobiotic mouse model for microbiome therapeutics. Exp. Mol. Med. *52*, 1383–1396. https://doi.org/10.1038/s12276-020-0473-2.

Parks, D.H., Chuvochina, M., Chaumeil, P.A., Rinke, C., Mussig, A.J., and Hugenholtz, P. (2020). A complete domain-to-species taxonomy for Bacteria and Archaea. Nat. Biotechnol. *38*, 1079–1086. https://doi.org/10.1038/s41587-020-0501-8.

Parks, D.H., Chuvochina, M., Waite, D.W., Rinke, C., Skarshewski, A., Chaumeil, P.A., and Hugenholtz, P. (2018). A standardized bacterial taxonomy based on genome phylogeny substantially revises the tree of life. Nat. Biotechnol. *36*, 996–1004. https://doi.org/10.1038/nbt.4229.

Parks, D.H., Imelfort, M., Skennerton, C.T., Hugenholtz, P., and Tyson, G.W. (2015). CheckM: assessing the quality of microbial genomes recovered from isolates, single cells, and metagenomes. Genome Res *25*, 1043–1055. https://doi.org/10.1101/gr.186072.114.

Parks, D.H., Rinke, C., Chuvochina, M., Chaumeil, P.A., Woodcroft, B.J., Evans, P.N., Hugenholtz, P., and Tyson, G.W. (2017). Recovery of nearly 8,000 metagenome-assembled genomes substantially expands the tree of life. Nat. Microbiol. *2*, 1533–1542. https://doi.org/10.1038/s41564-017-0012-7.

Pasolli, E., Asnicar, F., Manara, S., Zolfo, M., Karcher, N., Armanini, F., Beghini, F., Manghi, P., Tett, A., Ghensi, P., et al. (2019). Extensive unexplored human microbiome diversity revealed by over 150,000 genomes from metagenomes spanning age, geography, and lifestyle. Cell *176*, 649–662.e20. https://doi.org/10.1016/j.cell.2019.01.001.

Pedron, R., Esposito, A., Bianconi, I., Pasolli, E., Tett, A., Asnicar, F., Cristofolini, M., Segata, N., and Jousson, O. (2019). Genomic and metagenomic insights into the microbial community of a thermal spring. Microbiome *7*, 8. https://doi.org/10.1186/s40168-019-0625-6.

Poyet, M., Groussin, M., Gibbons, S.M., Avila-Pacheco, J., Jiang, X., Kearney, S.M., Perrotta, A.R., Berdy, B., Zhao, S., Lieberman, T.D., et al. (2019). A library of human gut bacterial isolates paired with longitudinal multiomics data enables mechanistic microbiome research. Nat. Med. *25*, 1442–1452. https://doi.org/10.1038/s41591-019-0559-3.

Price, M.N., Dehal, P.S., and Arkin, A.P. (2010). FastTree 2 – approximately maximum-likelihood trees for large alignments. PLoS One *5*, e9490. https://doi.org/10.1371/journal.pone.0009490.

Qin, J., Li, R., Raes, J., Arumugam, M., Burgdorf, K.S., Manichanh, C., Nielsen, T., Pons, N., Levenez, F., Yamada, T., et al. (2010). A human gut microbial gene catalogue established by metagenomic sequencing. Nature *464*, 59–65. https://doi.org/10.1038/nature08821.

R Core Team. (2020). R: A language and environment for statistical computing. R Foundation for Statistical Computing, Vienna, Austria https://www.R-project.org/.

Richardson, L.J., Rawlings, N.D., Salazar, G.A., Almeida, A., Haft, D.R., Ducq, G., Sutton, G.G., and Finn, R.D. (2019). Genome properties in 2019: a new companion database to InterPro for the inference of complete functional attributes. Nucleic Acids Res *47*, D564–D572. https://doi.org/10.1093/nar/gky1013.

Ridlon, J.M., Ikegawa, S., Alves, J.M.P., Zhou, B., Kobayashi, A., Iida, T., Mitamura, K., Tanabe, G., Serrano, M., De Guzman, A., et al. (2013). Clostridium scindens: a human gut microbe with a high potential to convert glucocorticoids into androgens. J. Lipid Res. *54*, 2437–2449. https://doi.org/10.1194/jlr.M038869.

Rosshart, S.P., Herz, J., Vassallo, B.G., Hunter, A., Wall, M.K., Badger, J.H., McCulloch, J.A., Anastasakis, D.G., Sarshad, A.A., Leonardi, I., et al. (2019). Laboratory mice born to wild mice have natural microbiota and model human immune responses. Science *365*, eaaw4361. https://doi.org/10.1126/science.aaw4361.

Rosshart, S.P., Vassallo, B.G., Angeletti, D., Hutchinson, D.S., Morgan, A.P., Takeda, K., Hickman, H.D., McCulloch, J.A., Badger, J.H., Ajami, N.J., et al. (2017). Wild mouse gut microbiota promotes host fitness and improves disease resistance. Cell *171*, 1015–1028.e13. https://doi.org/10.1016/j.cell.2017.09.016.

Schloss, P.D., Westcott, S.L., Ryabin, T., Hall, J.R., Hartmann, M., Hollister, E.B., Lesniewski, R.A., Oakley, B.B., Parks, D.H., Robinson, C.J., et al. (2009). Introducing mothur: open-source, platform-independent, community-supported software for describing and comparing microbial communities. Appl. Environ. Microbiol. *75*, 7537–7541. https://doi.org/10.1128/AEM.01541-09.

Seemann, T. (2014). Prokka: rapid prokaryotic genome annotation. Bioinformatics *30*, 2068–2069. https://doi.org/10.1093/bioinformatics/btu153.

Shaiber, A., and Eren, A.M. (2019). Composite metagenome-assembled genomes reduce the quality of public genome repositories. mBio *10*. e00725–e19. https://doi.org/10.1128/mBio.00725-19.

Silva, Y.P., Bernardi, A., and Frozza, R.L. (2020). The role of short-chain fatty acids from gut microbiota in gut-brain communication. Front. Endocrinol. *11*, 25. https://doi.org/10.3389/fendo.2020.00025.

Sokol, H., Pigneur, B., Watterlot, L., Lakhdari, O., Bermúdez-Humarán, L.G., Gratadoux, J.J., Blugeon, S., Bridonneau, C., Furet, J.P., Corthier, G., et al. (2008). Faecalibacterium prausnitzii is an anti-inflammatory commensal bacterium identified by gut microbiota analysis of Crohn disease patients. Proc. Natl. Acad. Sci. U. S. A. *105*, 16731–16736. https://doi.org/10.1073/pnas.0804812105.

Sonnenburg, J.L., and Bäckhed, F. (2016). Diet–microbiota interactions as moderators of human metabolism. Nature *535*, 56–64. https://doi.org/10.1038/nature18846.

Sorbara, M.T., Littmann, E.R., Fontana, E., Moody, T.U., Kohout, C.E., Gjonbalaj, M., Eaton, V., Seok, R., Leiner, I.M., and Pamer, E.G. (2020). Functional and genomic variation between human-derived isolates of Lachnospiraceae reveals inter- and intra-species diversity. Cell Host Microbe *28*, 134–146.e4. https://doi.org/10.1016/j.chom.2020.05.005.

Stappenbeck, T.S., and Virgin, H.W. (2016). Accounting for reciprocal host–microbiome interactions in experimental science. Nature *534*, 191–199. https://doi.org/10.1038/nature18285.

Steinegger, M., and Söding, J. (2017). MMseqs2 enables sensitive protein sequence searching for the analysis of massive data sets. Nat. Biotechnol. *35*, 1026–1028. https://doi.org/10.1038/nbt.3988.

Steinegger, M., and Söding, J. (2018). Clustering huge protein sequence sets in linear time. Nat. Commun. *9*, 2542. https://doi.org/10.1038/s41467-018-04964-5.

Surana, N.K., and Kasper, D.L. (2017). Moving beyond microbiome-wide associations to causal microbe identification. Nature *552*, 244–247. https://doi.org/10.1038/nature25019.

Szentirmai, É., Millican, N.S., Massie, A.R., and Kapás, L. (2019). Butyrate, a metabolite of intestinal bacteria, enhances sleep. Sci. Rep. *9*, 7035. https://doi.org/10.1038/s41598-019-43502-1.

Thomas, A.M., and Segata, N. (2019). Multiple levels of the unknown in microbiome research. BMC Biol *17*, 48. https://doi.org/10.1186/s12915-019-0667-z.

Tonkin-Hill, G., MacAlasdair, N., Ruis, C., Weimann, A., Horesh, G., Lees, J.A., Gladstone, R.A., Lo, S., Beaudoin, C., Floto, R.A., et al. (2020). Producing polished prokaryotic pangenomes with the Panaroo pipeline. Genome Biol *21*, 180. https://doi.org/10.1186/s13059-020-02090-4.

Consortium, UniProt (2021). UniProt: the universal protein KnowledgeBase in 2021. Nucleic Acids Res *49*, D480–D489. https://doi.org/10.1093/nar/gkaa1100.

Uritskiy, G.V., DiRuggiero, J., and Taylor, J. (2018). MetaWRAP—a flexible pipeline for genome-resolved metagenomic data analysis. Microbiome *6*, 158. https://doi.org/10.1186/s40168-018-0541-1.

Varghese, N.J., Mukherjee, S., Ivanova, N., Konstantinidis, K.T., Mavrommatis, K., Kyrpides, N.C., and Pati, A. (2015). Microbial species delineation using whole genome sequences. Nucleic Acids Res *43*, 6761–6771. https://doi.org/10.1093/nar/gkv657.

Wood, D.E., Lu, J., and Langmead, B. (2019). Improved metagenomic analysis with Kraken 2. Genome Biol *20*, 257. https://doi.org/10.1186/s13059-019-1891-0.

Wu, Y.W., Simmons, B.A., and Singer, S.W. (2016). MaxBin 2.0: an automated binning algorithm to recover genomes from multiple metagenomic datasets. Bioinformatics *32*, 605–607. https://doi.org/10.1093/bioinformatics/btv638.

Xiao, L., Feng, Q., Liang, S., Sonne, S.B., Xia, Z., Qiu, X., Li, X., Long, H., Zhang, J., Zhang, D., et al. (2015). A catalog of the mouse gut metagenome. Nat. Biotechnol. *33*, 1103–1108. https://doi.org/10.1038/nbt.3353.

Zerbino, D.R., and Birney, E. (2008). Velvet: algorithms for de novo short read assembly using de Bruijn graphs. Genome Res *18*, 821–829. https://doi.org/10.1101/gr.074492.107.

Zhu, J., Ren, H., Zhong, H., Li, X., Zou, Y., Han, M., Li, M., Madsen, L., Kristiansen, K., and Xiao, L. (2021). An expanded gene catalog of mouse gut metagenomes. mSphere *6*, e01119–e01120. https://doi.org/10.1128/mSphere.01119-20.

Zimmermann, M., Zimmermann-Kogadeeva, M., Wegmann, R., and Goodman, A.L. (2019a). Mapping human microbiome drug metabolism by gut bacteria and their genes. Nature *570*, 462–467. https://doi.org/10.1038/s41586-019-1291-3.

Zimmermann, M., Zimmermann-Kogadeeva, M., Wegmann, R., and Goodman, A.L. (2019b). Separating host and microbiome contributions to drug pharmacokinetics and toxicity. Science *363*, eaat9931. https://doi.org/10.1126/science.aat9931.

Zou, Y., Xue, W., Luo, G., Deng, Z., Qin, P., Guo, R., Sun, H., Xia, Y., Liang, S., Dai, Y., et al. (2019). 1,520 Reference genomes from cultivated human gut bacteria enable functional microbiome analyses. Nat. Biotechnol. *37*, 179–185. https://doi.org/10.1038/s41587-018-0008-8.

CellPress
OPEN ACCESS

STAR★METHOD

KEY RESOURCES TABLE

REAGENT or RESOURCE	SOURCE	IDENTIFIER
**Bacterial and virus strains**		
Bacterial isolates of the Mouse Culture Collection	This paper	https://github.com/BenBeresfordJones/MGBC/blob/main/MCC_deposition_accessions.xlsx
**Biological samples**		
Faecal samples from mouse colonies	This paper	N/A
**Critical commercial assays**		
FastDNA SPIN Kit for Soil	MPBio	Cat#6560200
MasterPure Complete DNA and RNA Purification Kit	Lucigen	Cat#MC85200
**Deposited data**		
Whole-genome sequencing data (Mouse Culture Collection)	This paper	SRA: PRJEB18589
Metagenomic sequencing data (mouse faeces)	This paper	SRA: PRJEB44285
Metagenomic sequencing data (mouse faeces)	This paper	SRA: PRJEB44286
Genome assemblies (Mouse Culture Collection)	This paper	SRA: PRJEB45232
Genome assemblies (representative MAGs)	This paper	SRA: PRJEB45234
Data for all genomes	This paper	https://github.com/BenBeresfordJones/MGBC
Custom MGBC Kraken2/Bracken database	This paper	https://zenodo.org/record/4836362
Global mouse metagenome cohort data	This paper	https://zenodo.org/record/4836362
MGBC protein catalogues	This paper	https://zenodo.org/record/4840586
Unified Human Gastrointestinal Genome (UHGG) collection	Almeida et al., 2021	http://ftp.ebi.ac.uk/pub/databases/metagenomics/mgnify_genomes/
Unified Human Gastrointestinal Protein (UHGP) catalogue	Almeida et al., 2021	http://ftp.ebi.ac.uk/pub/databases/metagenomics/mgnify_genomes/
Genome Reference Consortium Mouse Build 39 (GRCm39)	https://www.ncbi.nlm.nih.gov/bioproject/	NCBI-BioProject: PRJNA20689
Coliphage phi-X174 complete genome	https://www.ncbi.nlm.nih.gov/bioproject/	NCBI-BioProject: PRJNA14015
Mouse Gut Gene Catalog (MGCv1)	Xiao et al., 2015	http://gigadb.org/dataset/view/id/100114/token/mZlMYJIF04LshpgP
Integrated Mouse Gut Metagenomic Catalog (iMGMC)	Lesker et al., 2020	https://zenodo.org/record/3631711
Mouse Intestinal Bacterial Collection (miBC)	Lagkouvardos et al., 2016	SRA: PRJEB10572
Mouse Gut Microbial Biobank (mGMB)	Liu et al., 2020	SRA: PRJNA486904
**Oligonucleotides**		
Primer: Universal 16S rRNA Forward (7F): AGAGTTTGATYMTGGCTCAG	Browne et al., 2016	N/A
Primer: Universal 16S rRNA Reverse (1510R): CCTTCYGCAGGTTCACCTAC	Browne et al., 2016	N/A

(*Continued on next page*)

Continued		
REAGENT or RESOURCE	SOURCE	IDENTIFIER
Software and algorithms		
R version 4.0.2	R Core Team, 2020	https://www.r-project.org/
mothur version 1.46.1	Schloss et al., 2009	https://github.com/mothur/mothur
NCBI BLAST	Johnson et al., 2008	https://blast.ncbi.nlm.nih.gov
Velvet version 1.2	Zerbino and Birney, 2008	https://github.com/dzerbino/velvet
VelvetOptimiser version 2.2.5	N/A	https://github.com/tseemann/VelvetOptimiser
SSPACE version 2.1.1	Boetzer et al., 2011	https://github.com/nsoranzo/sspace_basic
GapFiller	Boetzer and Pirovano, 2012	http://www.baseclear.com/bioinformatics-tools/
Prokka version 1.14.5	Seeman, 2014	https://github.com/tseemann/prokka
MetaWRAP version 1.2.3	Uritskiy et al., 2018	https://github.com/bxlab/metaWRAP
KneadData version 0.7.3	The Huttenhower Lab	https://github.com/biobakery/kneaddata
Bowtie2 version 2.3.5	Langmead et al., 2019	https://github.com/BenLangmead/bowtie2
MetaSPAdes version 3.10.1	Nurk et al., 2017	https://github.com/ablab/spades
MEGAHIT version 1.1.1-2-g02102e1	Li et al., 2016	https://github.com/voutcn/megahit
MetaBAT2 version 2.9.1	Kang et al., 2019	https://bitbucket.org/berkeleylab/metabat
MaxBin 2.0 version 2.2.4	Wu et al., 2016	https://sourceforge.net/projects/maxbin/
CONCOCT version 0.4.0	Alneberg et al., 2014	https://github.com/BinPro/CONCOCT
CheckM version 1.1.2	Parks et al., 2015	https://github.com/Ecogenomics/CheckM
dRep version 2.5.4	Olm et al., 2017	https://github.com/MrOlm/drep
GTDB-Tk version 1.3-r95	Chaumeil et al., 2019	https://github.com/Ecogenomics/GTDBTk
Mash version 2.2.2	Ondov et al., 2016	https://github.com/marbl/Mash
FastANI version 1.3	Jain et al., 2018	https://github.com/ParBLiSS/FastANI
Panaroo version 1.2.4	Tonkin-Hill et al., 2020	https://github.com/gtonkinhill/panaroo
Kraken2 version 2.0.8	Wood et al., 2019	https://github.com/DerrickWood/kraken2
Bracken version 2.5.2	Lu et al., 2017	https://github.com/jenniferlu717/Bracken
zCompositions R package version 1.3.4	Palarea-Albaladejo and Martín-Fernández, 2015	https://cran.r-project.org/web/packages/zCompositions/
Vegan R package version 2.5-6	Oksanen et al., 2014	https://cran.r-project.org/web/packages/vegan/
MMseqs2 version 10-6d92c	Steinegger and Söding, 2018	https://github.com/soedinglab/MMseqs2
InterProScan version 5.39-77.0	Jones et al., 2014	https://github.com/ebi-pf-team/interproscan
Genome Properties version 2.0.1	(Richardson et al., 2019	https://www.ebi.ac.uk/interpro/genomeproperties/
EggNOG-mapper version 2.0.1	Huerta-Cepas et al., 2019	https://github.com/eggnogdb/eggnog-mapper
FastTree version 2.1.10	Price et al., 2010	http://www.microbesonline.org/fasttree/
IQ-TREE version 1.6.10	Nguyen et al., 2015	http://www.iqtree.org
Interactive Tree Of Life (iTOL) version 5.6.3	Letunic and Bork, 2021	https://itol.embl.de
Ape R package version 5.5	Paradis and Schliep, 2019	http://cran.r-project.org/package=ape
MGBC-Toolkit	This paper	https://github.com/BenBeresfordJones/MGBC-Toolkit
BLAST+ version 2.7.1	Camacho et al., 2009	https://blast.ncbi.nlm.nih.gov/Blast.cgi
CMseq version	Pasolli et al., 2019	https://github.com/SegataLab/cmseq
GUNC version 1.0.4	Orakov et al., 2021	https://github.com/grp-bork/gunc
Other		
European Nucleotide Archive (ENA)	Harrison et al., 2021	https://www.ebi.ac.uk/ena/browser/home
FastPrep-24 Classic bead beating grinder and lysis system	MPBio	Cat#6004500
RefSeq Release 205	O'Leary et al., 2016	https://www.ncbi.nlm.nih.gov/refseq/
UniProt	The UniProt Consortium (Sao Paulo)	https://www.uniprot.org/
Code for the MGBC	This paper	https://doi.org/10.5281/zenodo.5706242
Code for the MGBC-Toolkit	This paper	https://doi.org/10.5281/zenodo.5706246

RESOURCE AVAILABILITY

Lead contact

Further information and requests for resources, reagents and software should be directed to and will be fulfilled by the lead contact, Virginia A. Pedicord (vap33@cam.ac.uk).

Materials availability

Isolates of additional culturable species generated in this study are being deposited at the Leibniz Institute DSMZ-German Collection of Microorganism and Cell Cultures (DSMZ), and accession numbers are available under https://github.com/BenBeresfordJones/MGBC. Isolates generated in this study that have not been deposited at DSMZ will be made available without restriction upon request.

Data and code availability

- Raw sequencing data and genome assemblies for the isolates of the MCC have been deposited in the European Nucleotide Archive (ENA) under project accessions ENA: PRJEB18589 and ENA: PRJEB45232 respectively. Genome assemblies for the representative MAGs of the MGBC have been deposited in the ENA under project accession ENA: PRJEB45234. The MGBC Kraken2/Bracken database, protein catalogues and all genome assemblies and annotations generated in this study have been deposited at Zenodo and can be accessed via https://github.com/BenBeresfordJones/MGBC. DOIs are listed in the key resources table. Metagenomics sequences from 48 samples are deposited in the European Bioinformatics Institute-Sequence Read Archive (SRA) database under accessions ENA: PRJEB44285 and ENA: PRJEB44286. All datasets generated in this study are either included in Supplementary Tables or have been deposited on Zenodo and can be accessed via https://github.com/BenBeresfordJones/MGBC.
- The pipelines, workflows, and code to generate figures are available under https://github.com/BenBeresfordJones/MGBC. The MGBC-Toolkit is available at https://github.com/BenBeresfordJones/MGBC-Toolkit. All code produced by this project has been additionally deposited in Zenodo, and DOIs are provided in the STAR methods.
- Any additional information required to reanalyse the data reported in this paper is available from the lead contact upon request.

EXPERIMENTAL MODEL AND SUBJECT DETAILS

Faecal samples from mice

Mice were maintained under specific pathogen-free conditions at a Home Office-approved facility in accordance with the United Kingdom Animals (Scientific Procedures) Act of 1986. Faecal samples from 30 mice aged between 5 to 8 weeks were used to generate the Mouse Culture Collection. We used both male and female representatives from 10 different mouse colonies at the Wellcome Sanger Institute. Colony genotypes are detailed in Table S8. For shotgun metagenome sequencing, faecal samples were obtained from 48 8-week-old male C57BL/6N mice.

Bacterial culture

Fresh faecal samples were collected from mice into sterile 1.5mL Eppendorf tubes using aseptic technique. Sample processing and culturing were performed under anaerobic conditions (80% nitrogen, 10% carbon dioxide, 10% hydrogen) in a Don Whitley A95 anaerobic workstation. Faeces were homogenised in sterile, pre-reduced PBS (100mg/mL) and a 10-fold 1:10 dilution series performed. 200μL of each dilution was plated onto pre-reduced 140mm agar plates and incubated at 37°C. A range of agars were employed to maximise culturing yields and diversity (Table S8). After 2 days, individual colonies were picked and re-streaked onto fresh plates. This was then repeated until purity was achieved. Colonies were identified using 16S rRNA gene sequencing. Single bacterial colonies were scraped into 2mL screw cap tubes containing glass beads (acid-washed 425–600μm) and 500μL sterile PBS, and then physically lysed by 30 seconds shaking at speed 6.0 using a FastPrep Instrument (MPBio). After centrifugation at 14,000rpm for 5 minutes, 1μL of supernatant was taken to carry out a 16S PCR using the standard 7F and 1510R bacterial primers (Browne et al., 2016) and GoTaq Hot Start reagents (Promega). PCR products were sequenced by an external supplier (Eurofins Genomics) and mothur (Schloss et al., 2009) used to align the resulting sequences and create OTUs representing clusters of $\geq$97.8% sequence identity. For each OTU, a single sequence was taxonomically classified using NCBI BLAST (Johnson et al., 2008) and a single isolate selected for further culturing and whole-genome sequencing. 10mL of BHI or YCFA broth was inoculated for each new isolate identified and left to grow overnight. 500μL of the overnight culture was mixed with 500μL of 50% glycerol in a cryotube (performed in quadruplicate) and these were frozen at -80°C. The remaining overnight culture was centrifuged at 4,000rpm, and the cell pellet then washed with 10mL sterile PBS. For isolates that did not grow in broth, 2mL sterile PBS was pipetted onto mono-inoculated agar plates and colonies were dissolved using a bacterial scraper. Plate supernatants were then used in place of overnight cultures. Genomic DNA was extracted from the washed pellet using the 'MasterPure Complete DNA and RNA Purification Kit' (Lucigen). Genomic DNA was kept at 4°C until sequencing. While 276 strains were cultured to purity and sequenced, only 223 were subsequently recovered successfully from cryopreservation for banking at the Wellcome Sanger Institute and DSMZ.

METHOD DETAILS

Curation of public mouse gut-derived isolate genomes

To build a comprehensive genome collection, we curated 319 publicly available mouse-derived isolate genomes from the ENA, including the genomes of previously published mouse gut isolate collections (Lagkouvardos et al., 2019, 2016; Liu et al., 2020). As genome assemblies for the isolates of the miBC (Lagkouvardos et al., 2016) had not been made available, we assembled these from raw reads according to our standard genome processing pipeline. Only genomes that passed our quality control thresholds (n=288; "Quality control of genome assemblies") were included in the analyses for this study. The metadata for these public mouse isolate genomes are included in Table S1.

Curation of public human gut-derived bacterial genomes

To perform taxonomic and functional analyses between mouse and human gut bacterial species, we curated 204,939 non-redundant human gut microbial genomes from the Unified Human Gastrointestinal Genome (UHGG) catalogue (Almeida et al., 2021). We applied the same quality control and taxonomy assignment pipelines to the UHGG genomes as with the MGBC. In total, 100,456 non-redundant, high-quality human gut microbial genomes, representing 3,006 species, were curated for comparison with the MGBC. Metadata for these genomes are provided in Table S3.

Mouse gut metagenome cohort curation

To create a comprehensive mouse gut-derived metagenome catalogue for MAG synthesis and quantification of species global abundance and prevalence profiles, we utilised the Advanced Search functionality of the ENA (Harrison et al., 2021) to identify all WGS raw read samples with an NCBI Taxonomy metadata value of "mouse gut metagenome" (taxid: 410661; last accessed February 2021). In total, 8,418 samples were identified which were then manually assessed according to our exclusion criteria. We additionally performed a review of the available literature to identify further studies that our ENA search might have overlooked due to constraints with manual metadata entry. Samples were technically excluded if they were unpublished, i.e., where no publication listed the study accession number, or if they represented 16S rRNA amplicon sequencing datasets. Samples were biologically excluded if mice had been exposed to antibiotics, had an active gastrointestinal infection, or had received faecal microbiota transplantation derived from a non-mouse host. Furthermore, we additionally excluded samples that would disrupt the validity of species prevalence and abundance analyses, e.g., following enrichment for viral particles, or deriving from an ex-germfree mouse reconstituted with a simplified microbiota. Details of included and excluded studies are provided in Table S2. Of the included metagenomes, 64 were kept aside as independent samples for read classification analyses. Metadata for these samples are included in Table S2.

These publicly available samples were supplemented with 48 newly sequenced faecal samples from 8-week-old male C57BL/6N mice. In total, 2,913 metagenome samples were used to generate MAGs; data for these samples are supplied in Table S2. 2,446 samples yielded $\geq$1 MAG and were used to generate abundance and prevalence profiles of mouse gut microbial species. The metadata for these samples are provided in Table S2.

Whole-genome sequencing and assembly

Bacterial genomic DNA was sequenced using the Illumina Hi-Seq Ten platform at the Wellcome Sanger Institute with library fragment sizes of 200–300 bp, a read length of 150 bp and a target read depth of 100x. Annotated assemblies were produced using a previously described pipeline (Page et al., 2016). Briefly, multiple assemblies were generated from sequence reads using Velvet v1.2 (Zerbino and Birney, 2008) and VelvetOptimiser v2.2.5. An assembly improvement step was applied to the assembly with the best N50 (Page et al., 2016), and contigs were scaffolded using SSPACE v2.1.1 (Boetzer et al., 2011) and sequence gaps filled using GapFiller (Boetzer and Pirovano, 2012). Automated annotation was performed using Prokka v1.14.5 (Seemann, 2014).

Faecal sample collection and shotgun metagenomic sequencing

Faecal samples were collected directly from C57BL/6N mice using aseptic techniques, and immediately stored at -80°C until DNA extraction. DNA was extracted using the 'FastDNA Spin Kit for Soil' (MPBio) according to manufacturer's instructions and stored at -20°C until metagenomic sequencing. DNA samples were quantified using a Qubit 4 Fluorometer (Thermo Fisher), and samples with $\geq$100 ng DNA material proceeded to paired-end (2×150 bp) shotgun metagenomic sequencing on the HiSeq 4000 platform.

Workflow for metagenome-assembled genome (MAG) synthesis

MAGs were generated using a custom in-house pipeline that leveraged MetaWRAP v1.2.3 (Uritskiy et al., 2018) for single sample assembly, binning and bin refinement. First, metagenomes were quality controlled using KneadData v0.7.3 with default settings. Host reads were removed from samples using the GRCm39 reference genome and Bowtie2 v2.3.5 (Langmead et al., 2019). In addition, reads were aligned to the phi-X174 genome and removed. MetaSPAdes v3.10.1 (Nurk et al., 2017) was used for the assembly of paired-end samples. In line with other reports, runtime with MetaSPAdes was excessively long (>48 hours) for some samples (Kim et al., 2020). In these cases, and for samples with only unpaired reads, MEGAHIT v1.1.1-2-g02102e1 (Li et al., 2016) was used for assembly. To generate genome bins, we utilised the 'binning' module from MetaWRAP to run MetaBAT2 v2.9.1 (Kang et al., 2019), MaxBin 2.0 v2.2.4 (Wu et al., 2016) and CONCOCT v0.4.0 (Alneberg et al., 2014) in parallel on each sample. These bins were then consolidated and refined using the 'bin_refinement' module.

Quality control of genome assemblies

Both isolate genomes and MAGs were subjected to stringent genome quality criteria to be included in our analyses. Completeness, contamination, and genome fragmentation were estimated using CheckM v1.1.2 (Parks et al., 2015). Genome assemblies with ≥90% completeness, <5% contamination, maximum contig count ≤500 (Browne et al., 2016), maximum genome size ≤8 Mb (Zou et al., 2019), N50 ≥10,000 kb (Nayfach et al., 2019) and mean contig length ≥5 kb (Zou et al., 2019), were defined as high-quality genomes, in line with guidelines and previous studies (Bowers et al., 2017; Parks et al., 2017; Pasolli et al., 2019). Any isolate genome that did not meet these thresholds was excluded from analysis. For MAGs, we additionally defined medium-plus quality genomes as those assemblies with ≥50% completeness, <5% contamination and a quality score ≥50, where

$$QS = Completeness - (5 \times Contamination)$$

(Parks et al., 2017). This definition exceeds the medium-quality thresholds as defined by MIMAGs (Bowers et al., 2017). Only high-quality genomes were included in our analyses, unless otherwise indicated. Data for MAG yields from included samples are provided in Table S2.

Additional quality metrics were calculated to complement the contamination scores generated with CheckM. For the non-redundant, high-quality genomes of the MGBC genome chimerism was quantified using GUNC v1.0.4 (Orakov et al., 2021) and strain heterogeneity was assessed using CMseq v1.0.3 (Pasolli et al., 2019). Quality data for these genomes are provided in Table S3.

Taxonomic classification of genomes and species clustering

To remove redundancy from our collection of high-quality genomes, we used dRep v2.5.4 (Olm et al., 2017) to remove conspecific genomes that shared ≥99.9% ANI (options: -pa 0.999 –SkipSecondary). We taxonomically classified our genomes using the 'classify_wf' workflow from GTDB-Tk v1.3-r95 (Chaumeil et al., 2019). To generate species clusters for genomes that could not be assigned to a species-level taxonomy using GTDB-Tk, we clustered these genomes at ≥95% ANI using a two-step genomic distance analysis implemented by dRep (options: -comp 50 -con 5 -pa 0.9 -sa 0.95 -nc 0.6). Previously calculated quality data from CheckM were supplied for each genome with the –genomeInfo flag to reduce computation time. Genomes that shared ≥95% ANI at 0.6 alignment fraction were considered the same species (Jain et al., 2018; Nielsen et al., 2014; Varghese et al., 2015).

To determine genome representatives for each species cluster, we ranked each genome according to a modified quality score

$$mQS = Completeness - (5 \times Contamination) + log(N50)$$

and used the highest scoring genome from each species as the representative.

Determining prior cultured status of isolates

We inferred the cultured status of our isolates using a two-step approach. First, we compared our isolate genomes to the publicly available mouse-derived isolate genomes of the MGBC. Isolates were considered to be the same species if (1) they were designated as the same species by GTDB-Tk, or (2) they shared ≥95% ANI across an alignment fraction of ≥0.6, in the case of a non-species-level classification. Next, we searched our isolates that were not represented in the public mouse gut isolate collections against NCBI RefSeq release 205 (O'Leary et al., 2016) using Mash v2.2.2 (Ondov et al., 2016), after which the most similar RefSeq genome to each isolate was then compared using FastANI v1.3 (Jain et al., 2018). As RefSeq excludes metagenome-derived genomes, an isolate was designated as "previously uncultured" if it shared <95% ANI with the closest related genome.

Benchmarking binner performance for production of high-quality MAGs

We compared high-quality bins generated from a subset of 2,303 publicly available mouse gut metagenomes by MetaBAT2, MaxBin 2.0 and CONCOCT, as well as the consolidated bins generated by MetaWRAP refinement. Binner performance was compared across four metrics:

i. CheckM estimates of genome quality (completeness, contamination, quality score) across all high-quality bins
ii. Taxonomic coverage of high-quality bins (i.e., the number of species represented)
iii. Average quality score of high-quality bins on a per species basis
iv. Core genome conservation by high-quality bins

Akkermansia muciniphila (Am), *Bifidobacterium globosum* (Bg), *Ligilactobacillus murinus* (Lm), and *Lactobacillus johnsonii* (Lj) were selected for comparing the conservation of the core genome between binners as these species ranked among the top 10 most commonly binned species across all four binning tools, and each have ≥50 isolate genomes with which to build a robust baseline core genome. In addition, these species represent three phyla, reducing the potential for any taxonomic bias. Isolate genomes were compiled from RefSeq and the MGBC, and high-quality isolate genomes that were designated as the correct species by GTDB-Tk were annotated using Prokka v1.14.5 with default settings. For each species, Panaroo v1.2.4 (Tonkin-Hill et al., 2020) was used to build an 'isolate-only' core genome and an 'isolate+bins' core genome for each binner using the following options: −clean-mode strict –core_threshold 0.99. The number of isolate genomes used for each species was as follows: Am, 136; Bg, 62; Lm, 58; Lj, 54. For each species, a standardised number of subsampled bins was used to build the 'isolate+bins' core genome for each binner: Am, 90; Bg, 35; Lm, 150; Lj, 60. To quantify core genome conservation, 100 iterations of bin subsampling and core genome analysis

were performed for each binner, and the core genome size distribution was calculated as a percentage of the 'isolate-only' core genome.

Comparison to other mouse microbiota resources

The MAGs of the iMGMC and co-abundance gene groups (CAGs) from Xiao et al. (2015) (MGCv1) were accessed and processed according to the quality control and taxonomic assignment protocols above. 8,509 MAGs of the iMGMC were defined as high-quality genomes and used for benchmarking the MGBC. Comparison of this resource to the MGBC consisted of three stages: taxonomic coverage, genome quality, and metagenome coverage. Shared and unique species were identified between the two collections using GTDB-Tk and dRep as performed for the MGBC alone. For each shared species, the quality score was compared between the representative genome of each collection.

To assess metagenomic read classification performance, custom Kraken2 (Wood et al., 2019) databases were built for all high-quality genomes (MGBC, iMGMC) and medium-plus quality genomes (mq MGBC, mq iMGMC) of the MGBC and iMGMC. As CAGs cannot be defined as high-quality due to failure to meet minimum genome fragmentation criteria (Bowers et al., 2017), a custom Kraken2 database for the 239 CAGs defined as medium-plus quality was built for comparison (MGCv1). In addition, custom Kraken2 databases were built for the post-qc isolate genomes of the miBC (Lagkouvardos et al., 2016) (n=43), the mGMB (Liu et al., 2020) (n=100), all publicly available mouse-derived isolates (Public, n=288), the Mouse Culture Collection (MCC, n=276), all mouse isolates (MCC+Public, n=564), and representative genomes of the high-quality species of the Unified Human Gastrointestinal Genome (Human, n=3,006). The standard Kraken2 database for all NCBI genomes (accessed 2nd December 2020) was also included. 64 independent, post-qc mouse gut metagenome samples that had not been included in the generation of the MGBC were analysed with the different Kraken2 databases and percentage read classification was utilised as a proxy for database efficiency. The metadata for these samples are included in Table S2.

Construction of phylogenetic trees

Maximum likelihood phylogenetic trees were built *de novo* from protein sequence alignments of 120 core bacterial genes generated by the GTDB-Tk 'align' module. The phylogenetic trees of the Mouse Culture Collection and the MGBC representative genomes were built using FastTree v2.1.10 (Price et al., 2010) with default settings (BLOSUM45 matrix; JTT+CAT model). IQ-TREE v1.6.10 (Nguyen et al., 2015) was used with default settings to build a phylogenetic tree of representative genomes for 3,006 human-derived and 1,094 mouse-derived bacterial species. LG+F+R10 was identified as the best fit protein substitution model based on the Bayesian information criterion (Kalyaanamoorthy et al., 2017). Trees were visualised using Interactive Tree Of Life v5.6.3 (Letunic and Bork, 2021).

Metagenome classification and analysis

For analysis of mouse gut shotgun metagenome samples, taxonomic classification was performed using Kraken2 v2.0.8 (Wood et al., 2019) and Bracken v2.5.2 (Lu et al., 2017). To enable species-resolved metagenomic analyses, we built a custom Kraken2/Bracken database (options: -k 31 -l 150) with the 26,640 high-quality genomes of the MGBC using a custom GTDB taxonomy (Parks et al., 2020, 2018). Only post-qc metagenomes that were of sufficient read depth to generate MAGs were used in metagenomic analyses (n=2,446). The resulting Bracken outputs were compiled and analysed with R v4.0.2 (R Core Team, 2020). To calculate prevalence, a threshold of 0.01% assigned classified reads was used to define presence of a species in a sample.

Due to the compositional nature metagenome analyses, we determined the Aitchison distances between samples (Aitchison, 1992). We performed Bayesian-multiplicative treatment of count zeros (Martín-Fernández et al., 2015) using the zCompositions v1.3.4 R package (Palarea-Albaladejo and Martín-Fernández, 2015) and transformed data using a center log-ratio transformation. Finally, the Euclidean distances of samples were determined using the vegan R package v2.5-6 (Oksanen et al., 2014). To assess the ability of metadata variables to explain variance in microbial communities of the mouse microbiome, we applied the 'adonis' function from vegan to calculate the Permutational Multivariate Analysis of Variance of the Aitchison distance matrices using 999 permutations. The PERMANOVA summary statistics are provided in Table S5.

For institute analyses, samples from "control" C57BL/6 mice were curated that were 1) faecal samples, 2) from wildtype mice, 3) not exposed to a wild mouse microbiota, and 4) fed only a regular chow diet. Only institutes that were represented by ≥ 10 samples were compared. Center log-ratio transformation of the data was performed and a heatmap generated using the pheatmap R package v1.0.12 for the top 20 most abundant species of these samples against institute. For laboratory vs wild analyses, 65 samples from 'wild' gut microbiotas were compared against 1,065 samples from control 'laboratory' microbiotas. Hybrid microbiotas, where wild and laboratory mice were crossed, were excluded from these analyses.

Taxonomic comparison of the mouse and human gut microbiotas

For taxonomic comparison, species were considered shared between the UHGG and the MGBC if they were annotated as the same species by GTDB-Tk, or, if they could not be assigned at a species-level, the representative genomes shared $\geq 95\%$ ANI across a minimum alignment fraction of 0.6.

Pangenome synthesis and functional annotation

To generate species pangenomes for functional annotation, we first concatenated 76,937,350 pre-clustered (100% sequence identity) proteins derived from the 100,456 non-redundant, high-quality genomes of the UHGG with 67,768,723 predicted proteins from

the 26,640 non-redundant, high-quality genomes of the MGBC, and performed protein clustering using the 'linclust' function from MMseqs2 v10-6d92c (-c 0.8 –cov-mode 1 –cluster-mode 2 –kmer-per-seq 80) (Steinegger and Söding, 2018, 2017). Proteins were clustered at 100%, 90%, 80% and 50% sequence identity.

Next, we generated species pangenomes by concatenating all non-redundant (90% sequence identity) protein-coding sequences from member genomes. Pangenomes were then functionally annotated using both InterProScan v5.39-77.0 (Jones et al., 2014) and EggNOG-mapper v2.0.1 (Huerta-Cepas et al., 2017).

To assess global functional overlap between host gut microbiotas, we calculated the number of InterPro (IPR) protein families and KEGG orthology (KO) groups that were shared in total between human and mouse pangenomes.

Taxonomic and functional distance analyses

Taxonomic distances were calculated from the human-mouse representative genome phylogeny ("Construction of phylogenetic trees"). Phylogenetic branch lengths between the representative genomes of each human and mouse species were calculated using the 'cophenetic.phylo' function from ape v5.5 (Paradis and Schliep, 2019).

To maximise the resolution of functional comparisons, functional annotations from multiple schemes – including InterPro, KEGG (Kanehisa et al., 2017), MetaCyc (Caspi et al., 2016), CAZy (Lombard et al., 2014), Reactome (Jassal et al., 2020), and Gene Ontology (Ashburner et al., 2000) – were considered together (all functions) in the context of each species pangenome. To facilitate functional distance analyses, pangenome-feature matrices were constructed where each functional annotation was scored according to the fraction of genomes per pangenome that were annotated with that feature. Inter-pangenomic functional distances were then calculated using the Jaccard Index.

A global comparison of the interspecies taxonomic and functional relationships was performed using a Mantel test. The taxonomic and functional distance matrices were ordinated using the 'cmdscale' function, and the two most dominant principal coordinates were visualised using the R packages ggpubr v0.4.0 and ggplot2 v3.3.5.

Functional comparison of *Phocaeicola* species

To identify the functional pathways that underpin the divergence between human and mouse *Phocaeicola dorei* (PdH, PdM) and the relative similarity of human *P. dorei* and mouse *P. vulgatus* (PvM), InterProScan v5.39-77.0 and Genome Properties v2.0.1 (Richardson et al., 2019) were run on all genomes for these species (PdH, n=1,954; PdM, n=15; PvM, n=177). The tabular outputs were concatenated and the proportion of 'complete pathway'-encoding genomes for each species was calculated for each property. The proportion of property-encoding genomes was then compared between each species pair using two-proportion z-tests with Yates' continuity correction. The data from these analyses are provided in Table S10.

Drug metabolism analyses

The protein sequences of genes validated for drug metabolism by the human gut microbiota from four independent studies (Table S7) were accessed via UniProt (The UniProt Consortium, 2021). Each gene was queried against a UHGG-MGBC combined protein catalogue (pre-clustered at 100% sequence identity) using BLAST+ v2.7.1 (Camacho et al., 2009). The 'hm_blast' module of the MGBC-Toolkit (https://github.com/BenBeresfordJones/MGBC-Toolkit) was utilised for these analyses. Hits with $\geq$95% sequence identity were considered functionally equivalent, and the genomes of origin for each hit were identified. For *bt_4096*, additional hits with sequence identity down to 50% were considered.

Butyrate synthesis analyses

The 'feature_search' module of the MGBC-Toolkit was applied to search bacterial pangenomes for the IPR family identifiers of the terminal pathways of butyrate synthesis: IPR023990 (BCOAT), IPR011245 (BUK), IPR014079 (PTB). Only species encoding both BUK and PTB were considered as butyrate producers using the PTB/BUK terminal pathway. The fraction of genomes per pangenome that were predicted to encode terminal pathway genes was calculated and based on these data a threshold of 70% was used to define butyrate-producing species.

Validating butyrate terminal pathway predictions using isolate cultures

From the functional annotations of their genomes, isolates from the Mouse Culture Collection were identified that encoded either the BCOAT pathway, the PTB/BUK pathway or neither. For each pathway, three isolates that ranked in the top 30 most abundant predicted butyrate-producing species according to the global mouse gut metagenome catalogue curated above ("Metagenome classification and analysis") were selected for culturing. Isolates for known butyrate-producing species of the human gut microbiota (*Agathobacter rectalis*, *Coprococcus eutactus_A*) were included as positive controls, and an isolate from the MCC that lacked predicted butyrate terminal pathway genes (*Lachnospiraceae_NOV* MGBC000113) was included as a negative control.

Under anaerobic conditions and using pre-reduced reagents, isolates were streaked onto YCFA (Duncan et al., 2002) agar and single colonies picked into 10 mL YCFA broth. Broth cultures were incubated at 37°C for 48 hours. Culture turbidity and pH were measured to confirm growth, and 16S rRNA gene sequencing was performed to check for contamination. Bacterial cultures were centrifuged at 3,600rpm for 5 minutes to pellet cells. The supernatant was pipetted off and sterile-filtered before being immediately stored at -80°C until analysis. These experiments were performed in triplicate.

Short chain fatty acid quantitation by GC-MS

Bacterial media samples (100 μL) were extracted using 400 μL methanol containing acetate-d3, propionate-d5, butyrate-d7 and valerate-d9 as internal standards (Cambridge Isotope Laboratories). After vortexing, samples were incubated at -80°C for >1 h to promote protein precipitation, then centrifuged for 20 min at 20,000g at 4°C. 100 μL of the resulting supernatant was added to 100 μL of 100 mM borate buffer (pH 10). Subsequently, 400 μL of 100 mM pentafluorobenzyl bromide (Thermo Scientific) diluted in acetonitrile (Fisher) and 400 μl of cyclohexane (Acros Organics) were added and reaction vials were sealed. Samples were derivatised by heating to 65°C for 1 h with agitation, then cooled to room temperature and centrifuged at 2,000g for 2 min to promote phase separation. 100 μL of the cyclohexane (upper) phase was transferred to a fresh autosampler vial and diluted 1:100 with cyclohexane prior to analysis. Gas chromatography-mass spectrometry (GC-MS) was performed using an Agilent 7890A gas chromatograph and Agilent 5975C MS detector operating in negative chemical ionisation mode. A 1μL splitless injection was made onto a VF-1701ms column (30 m × 0.25 mm, 0.25 μm; Agilent Technologies). Helium (1.2 mL/min) was the carrier gas and methane (2 mL/min) was used as the chemical ionisation reagent gas. For SCFA quantification, the peak areas of acetate (m/z 59) and propionate (m/z 73) were normalized to acetate-d3 (m/z 62) and propionate-d5 (m/z 78) internal standards respectively; the C4 compounds butyrate and isobutyrate (m/z 87) were normalized to butyrate-d7 (m/z 94) and the C5 compounds 2-methylbutyrate, valerate and isovalerate (m/z 101) were normalized to valerate-d9 (m/z 110). Calibrator and quality control (QC) samples were prepared in borate buffer and derivatised using the same procedure covering the range 0.05–125 mM. All data analyses were performed with Agilent MassHunter quantitative analysis software (version 10.1, Agilent Technologies) and QC samples were confirmed to be within ±15% accuracy. Butyrate production data are provided in Table S9.

QUANTIFICATION AND STATISTICAL ANALYSIS

All statistical analyses were performed using R v4.0.2. Statistical significance was verified using tests as reported in the text, STAR methods and figure legends. Tests for correlation utilised Pearson coefficients unless otherwise stated. A p- or q-value $\leq$0.05 was considered statistically significant. Experimental ‘n’ is reported throughout the results and methods, as well as in figure legends. Summary statistics, including measures of center and dispersion, are reported in the results section where appropriate. Graphs were generated using the ggpubr and ggplot2 packages in R v4.0.2.

Cell Host & Microbe, Volume *30*

Supplemental information

The Mouse Gastrointestinal Bacteria Catalogue enables translation between the mouse and human gut microbiotas via functional mapping

Benjamin S. Beresford-Jones, Samuel C. Forster, Mark D. Stares, George Notley, Elisa Viciani, Hilary P. Browne, Daniel J. Boehmler, Amelia T. Soderholm, Nitin Kumar, Kevin Vervier, Justin R. Cross, Alexandre Almeida, Trevor D. Lawley, and Virginia A. Pedicord

Supplemental information:
The Mouse Gastrointestinal Bacteria Catalogue enables translation between the mouse and human gut microbiotas via functional mapping
Beresford-Jones et al.

Contents:
Supplementary Figures 1-4
Supplementary Table 5

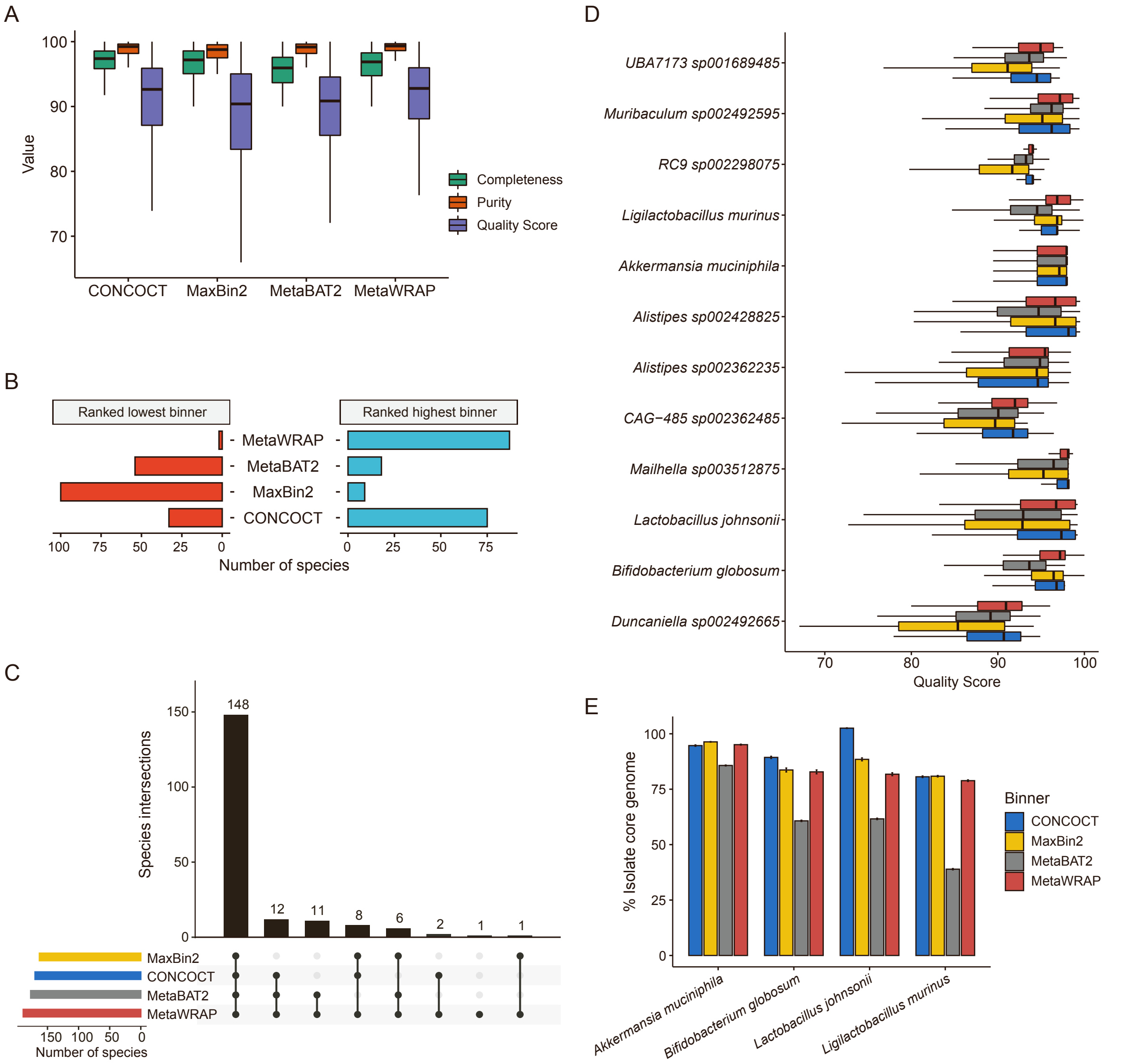

Supplementary Figure 1: Comparison of common binners for MAG synthesis, related to STAR methods. a) Quality scores of high-quality and medium-quality bins generated using the single binners MetaBAT2, MaxBin2, CONCOCT and hybrid bins from MetaWRAP (combined bins from all three single binners). b) Bars represent the number of species for which each binner generated the lowest quality bins ("Ranked lowest binner", red), and the highest quality ("Ranked highest binner", blue). c) UpSet plot illustrating the number of species represented by high-quality bins generated by each binner, and the species intersections between binners. d) MAG quality scores for the 12 most commonly binned species. e) Core genome size of commonly binned species when utilising different binners. Data represent the core genome size of MAG+isolate core genomes, compared to isolates alone. *A. muciniphila*, 100 iterations of 90 MAGs per binner and 136 isolate genomes; *B. globosum*, 100 iterations of 35 MAGs per binner and 62 isolate genomes; *L. johnsonii*, 100 iterations of 60 MAGs per binner and 54 isolate genomes; *L. murinus*, 100 iterations of 150 MAGs per binner and 58 isolate genomes. Aside from the medium quality MAG data in (a), only high-quality MAGs were used in these analyses.

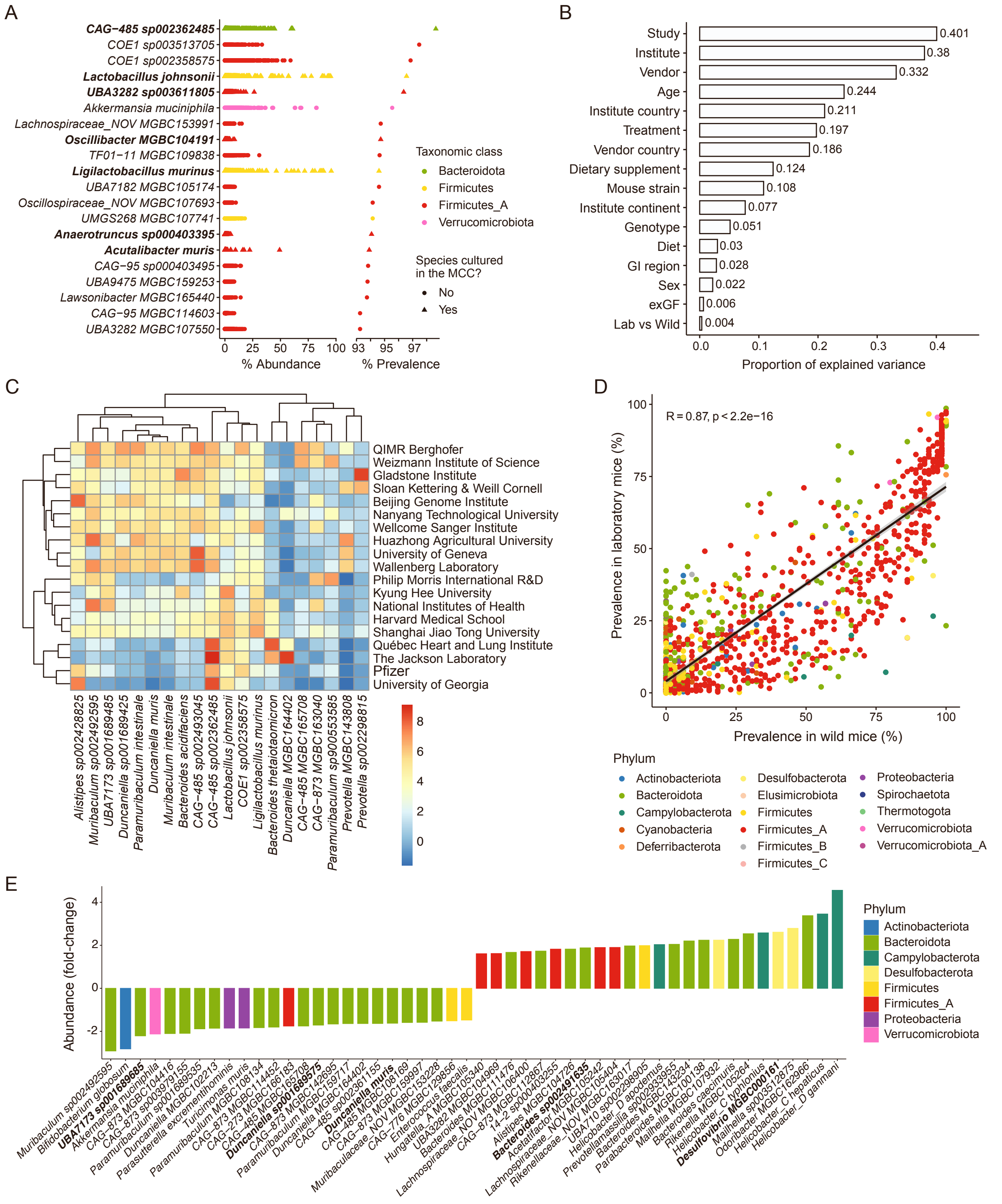

Supplementary Figure 2: The mouse gut microbiota between institutes and wild mice, related to STAR methods. a) Abundance and prevalence profiles of the 20 most prevalent species of the mouse gut microbiota across 2,446 samples. A species was determined as present in a sample if it was assigned ≥0.01% of classified reads. Point colour represents taxonomic phylum and point shapes and boldface labels indicate whether a species has been cultured as part of the MCC (triangles, bold) or not (circles). b) Proportion of variance explained (R2) by variables in the metadata using a permutational analysis of variance (PERMANOVA). All analyses were run with 999 permutations. Statistics for the PERMANOVA are provided in Table S7. c) Heatmap showing abundance of the top 20 most abundant species of the mouse microbiota across different institutes. Analyses include faecal samples from wildtype C57BL/6 “control” mice fed chow diets (n=432). Data are centre log-ratio

normalised read fractions, following Bayesian-multiplicative replacement of count zeros. d) Scatter plot comparing prevalence of species between untreated laboratory (n=1,065) and wild (n=65) mouse gut microbiotas. Each datapoint represents a mouse, and colour represents taxonomic phylum. Black line and shadow indicate linear regression line with 95% confidence interval (r=0.87, p<2.2x10-16). e) Fold-change in mean abundance of species in wild mice compared to untreated laboratory mice. Positive coefficient indicates enrichment in wild mice. Bar colour represents taxonomic phylum.

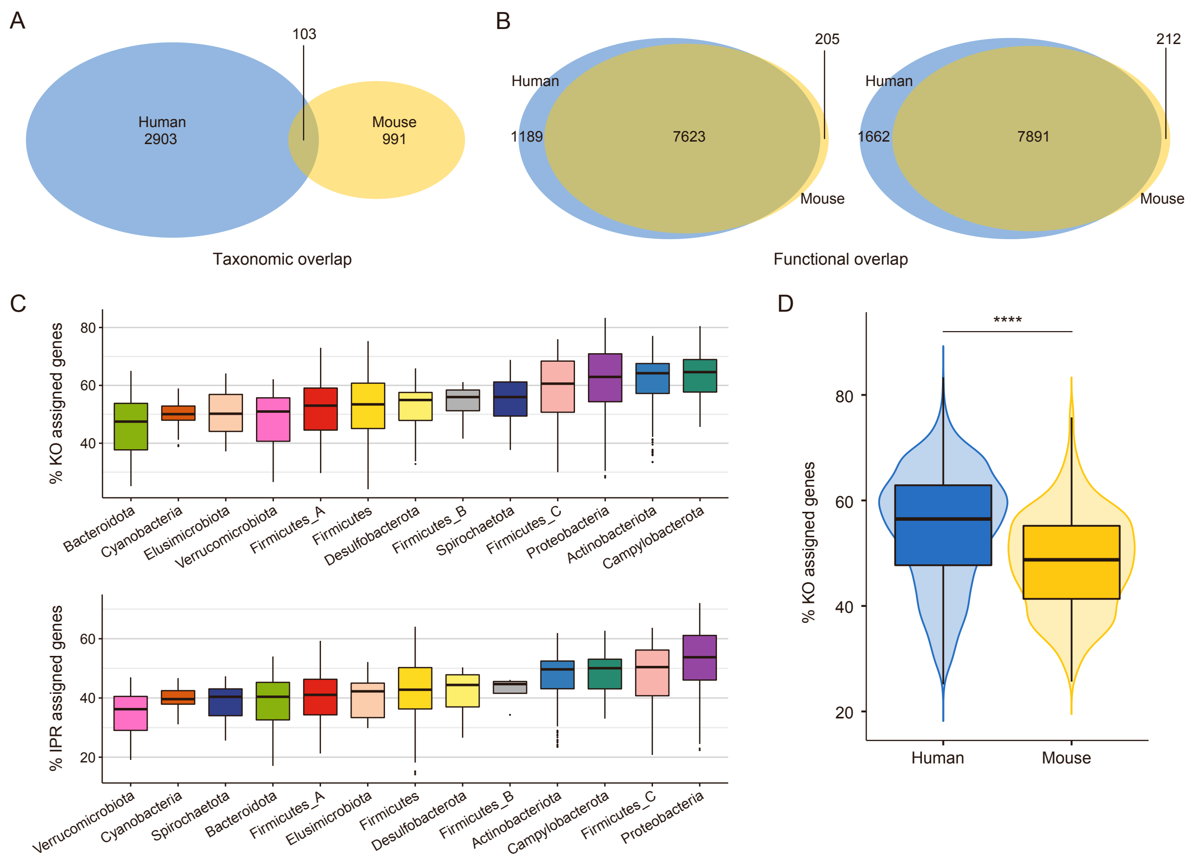

Supplementary Figure 3: Taxonomic and functional analyses of the human and mouse gut microbiotas, related to Figure 4. a) Venn diagram illustrating species sharing between human and mouse microbiotas. b) Venn diagrams illustrating the functional overlap of InterPro protein families (IPR; left) or KEGG Orthology (KO) groups (right) between all human (blue) and mouse (yellow) gut bacterial species. c) Functional annotation efficiency of bacterial pangenomes by taxonomic phylum for KO groups (top) and IPR families (bottom). Data represent the percentage of predicted protein-coding genes of each pangenome that could be assigned to an IPR or KO, coloured by phylum. d) Functional annotation efficiency of KO groups by host organism. A Wilcoxon signed-rank test was used to calculate statistical significance, ****P < 0.0001.

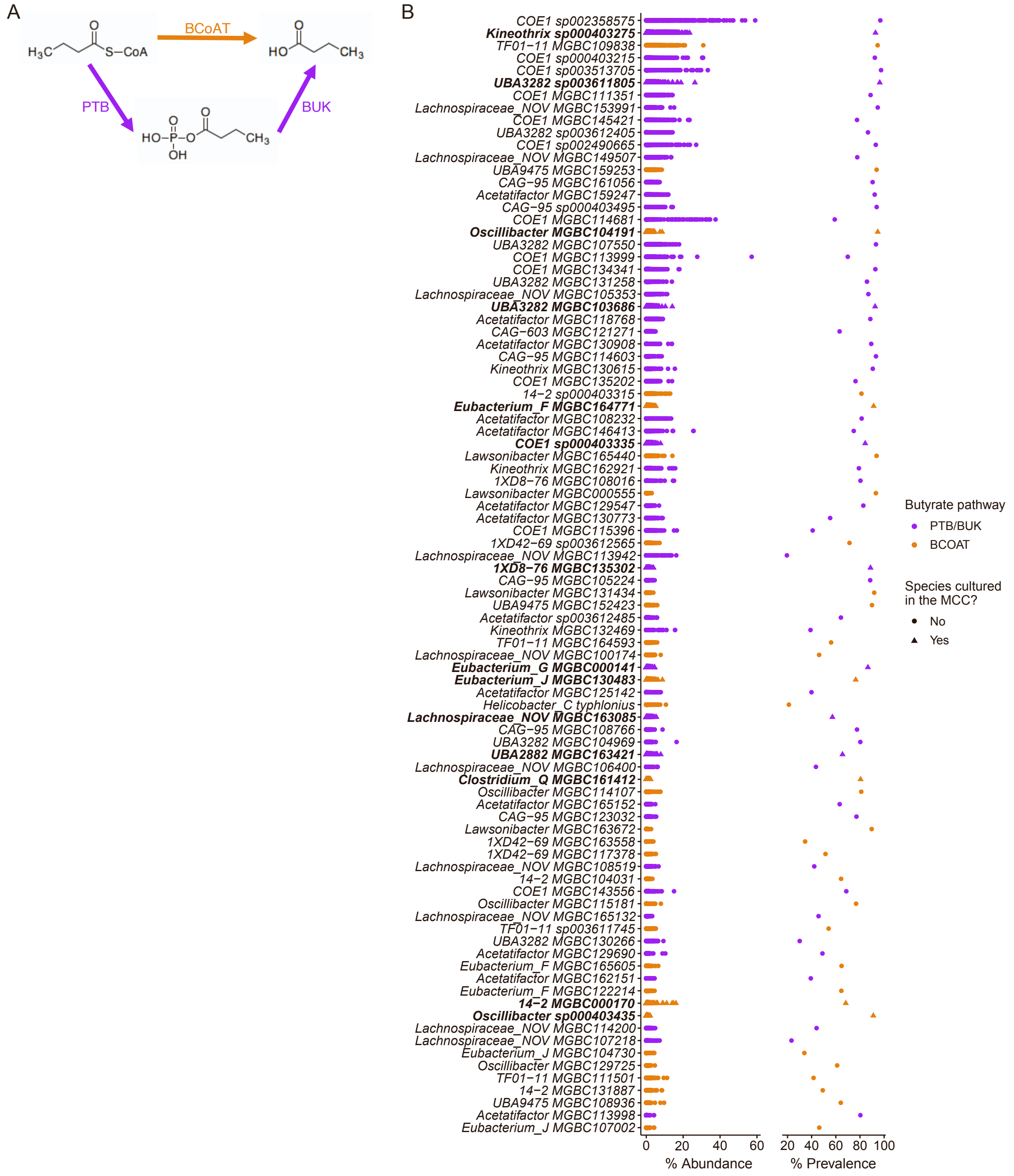

Supplementary Figure 4: Butyrate metabolism by species of the mouse gut microbiota, related to Figure 6. a) Schematic of the terminal pathways of butyrate synthesis by the gut microbiota. Butyrate CoA-transferase (BCoAT; orange); butyrate phosphotransferase/butyrate kinase (PTB/BUK; purple). b) Abundance and prevalence profiles of the 90 most abundant predicted butyrate producing species of the mouse gut microbiota. Point colour represents predicted encoded terminal butyrate pathway. Point shapes and boldface labels indicate whether a species has been cultured as part of the MCC (triangles, bold) or not (circles).

Supplementary Table 5: PERMANOVA statistics, related to STAR methods.
PERMANOVA comparing the extent to which factors in sample metadata explain variance between metagenomic samples.

Metadata factor	DegreesFreedom	SumsOfSquares	MeanSquares	F.Model	R2	P_value	Explanation of factor/variable:
Study	74	2055298	27774.3	21.42	0.40067	0.001	Study accession.
Institute	62	1949409	31442.1	23.56	0.38002	0.001	Experimental institute i.e., location of mice at time of sampling.
Vendor	57	1688944	29630.6	20.646	0.33217	0.001	Vendor's name with region if relevant e.g., Jackson, US vs Jackson, Denmark.
Age	54	1084273	20079.1	12.347	0.24407	0.001	Age at time of sampling.
Institute country	16	1074085	67130	40.29	0.21124	0.001	Country of the institute.
Treatment	58	1010917	17429.6	10.102	0.19728	0.001	What treatments did the mice receive e.g., control, high fat diet, specific drug treatment.
Vendor country	14	932012	66572	38.714	0.18555	0.001	Country of the vendor.
Dietary Supplement	30	635939	21198	11.392	0.1241	0.001	Dietary supplement, including in the drinking water e.g., glucose, NSAIDs, or DSS.
Mouse strain	20	553619	27681	14.671	0.10806	0.001	Strain of mice e.g., C57BL/6J vs C57BL/6NTac
Institute continent	3	394842	131614	67.88	0.07697	0.001	Continent of the institute.
Genotype	17	263389	15493.5	7.7309	0.05141	0.001	Genotype: wildtype, or specific knockout e.g., IL-10 deficient.
Diet	10	152023	15202.3	7.437	0.03	0.001	Diet: chow, high fat, low fibre, etc.
Sample source/GI location	10	143611	14361.1	7.0171	0.02825	0.001	Sampling location e.g., faeces, caecum, small intestine.
Sex	1	94158	94158	45.948	0.02201	0.001	Sex of mice.
exGF	1	32620	32620	15.641	0.00636	0.001	Endogenous (SPF) or reconstituted (ExGF) microbiota.
Lab vs Wild	1	18098	18098.1	8.6532	0.00353	0.001	Laboratory vs wild mice, reflecting their faecal origins e.g., in the case of ExGF mice

Technical features:	Permutation:	Free
	Number of permutations:	999
